# Supplementary material for: Centromere DNA Destabilizes H3 Nucleosomes to Promote CENP-A Deposition during the Cell Cycle
Source: Curr Biol. 2018 Dec 17;28(24):3924–3936.e4. doi: 10.1016/j.cub.2018.10.049 (PMC6303189; doi:10.1016/j.cub.2018.10.049)
Supplement: Document S1. Figures S1–S5 and Tables S1 and S2 [file mmc1.pdf]

Current Biology, Volume *28*

Supplemental Information

Centromere DNA Destabilizes H3 Nucleosomes

to Promote CENP-A Deposition during the Cell Cycle

Manu Shukla, Pin Tong, Sharon A. White, Puneet P. Singh, Angus M. Reid, Sandra Catania, Alison L. Pidoux, and Robin C. Allshire

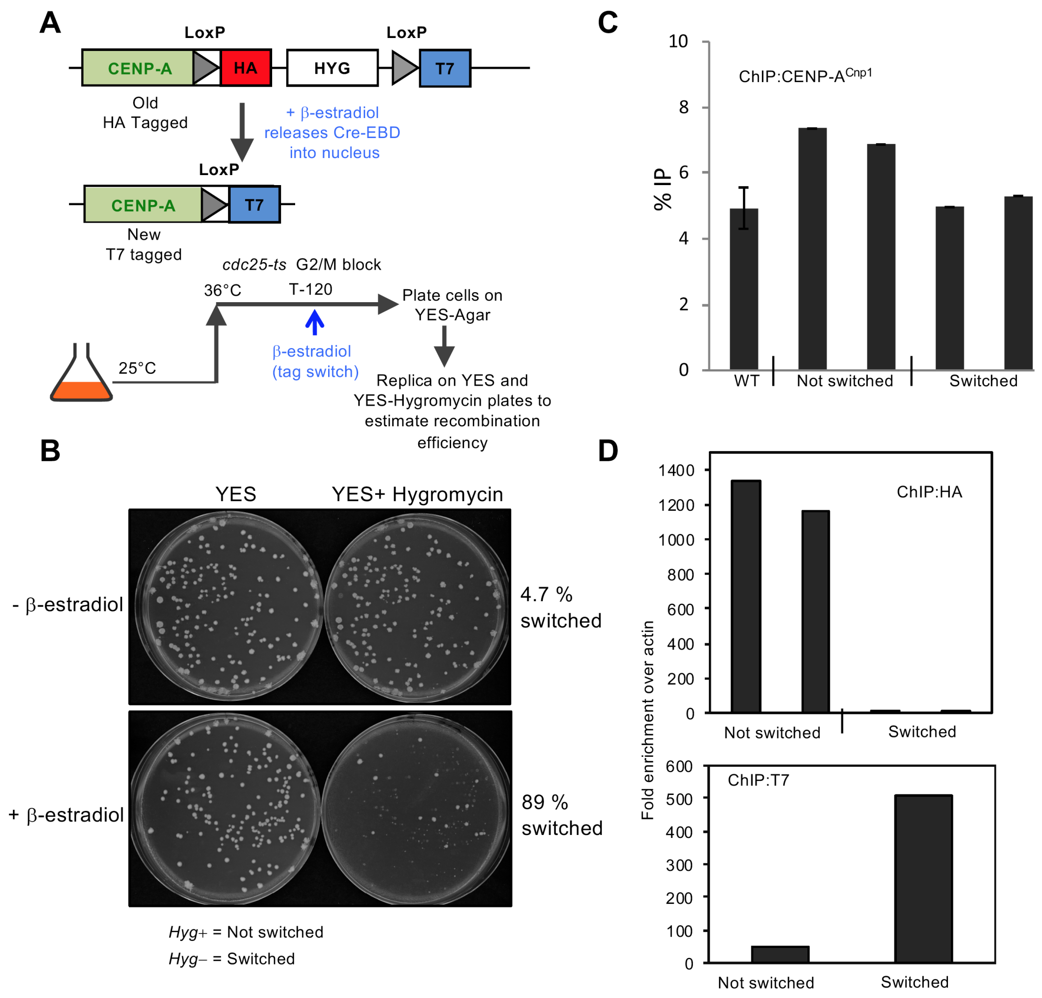

Figure S1. Validation of Recombination Induced Tag Exchange (RITE) system in *S. pombe*. Related to figure 1.

(A) Schematics of the CENP-A^{Cnp1} RITE experiment. *cdc25-22* cells were blocked in G2 and tag switch was induced by addition of β-estradiol as described in Figure 1A. After 2 hours of β-estradiol treatment, cells were plated on non-selective YES agar plates. Cre-EBD induced recombination results in tag switch accompanied by loss of *hygMX6* marker. Colonies were replica plated on YES agar plates with or without hygromycin to ascertain efficiency of tag switch.

(B) Representative experiment showing efficiency of tag switching. Percentage of *hyg*$^{+}$ colonies with or without β-estradiol treatment is shown.

(C) Centromeric CENP-A^{Cnp1} levels are not affected by presence of C-terminal RITE tags. CENP-A^{Cnp1} ChIP was performed on Non-switched (*hyg*$^{+}$) and switched (*hyg*$^{-}$) RITE tagged cell colonies (from B). Untagged wild type strain was used as control (Error bar represents mean ±SD, n=3). Y axis shows percentage immunoprecipitation values at central core 2 (*cc2*).

(D) Verification of tag switch by ChIP. Non-switched (*hyg*$^{+}$) and switched (*hyg*$^{-}$) RITE tagged cell colonies (from B) were grown in liquid culture and ChIPs for HA and T7 epitopes were performed. ChIP results show loss of HA tag (top panel) and presence of T7 tag after RITE.

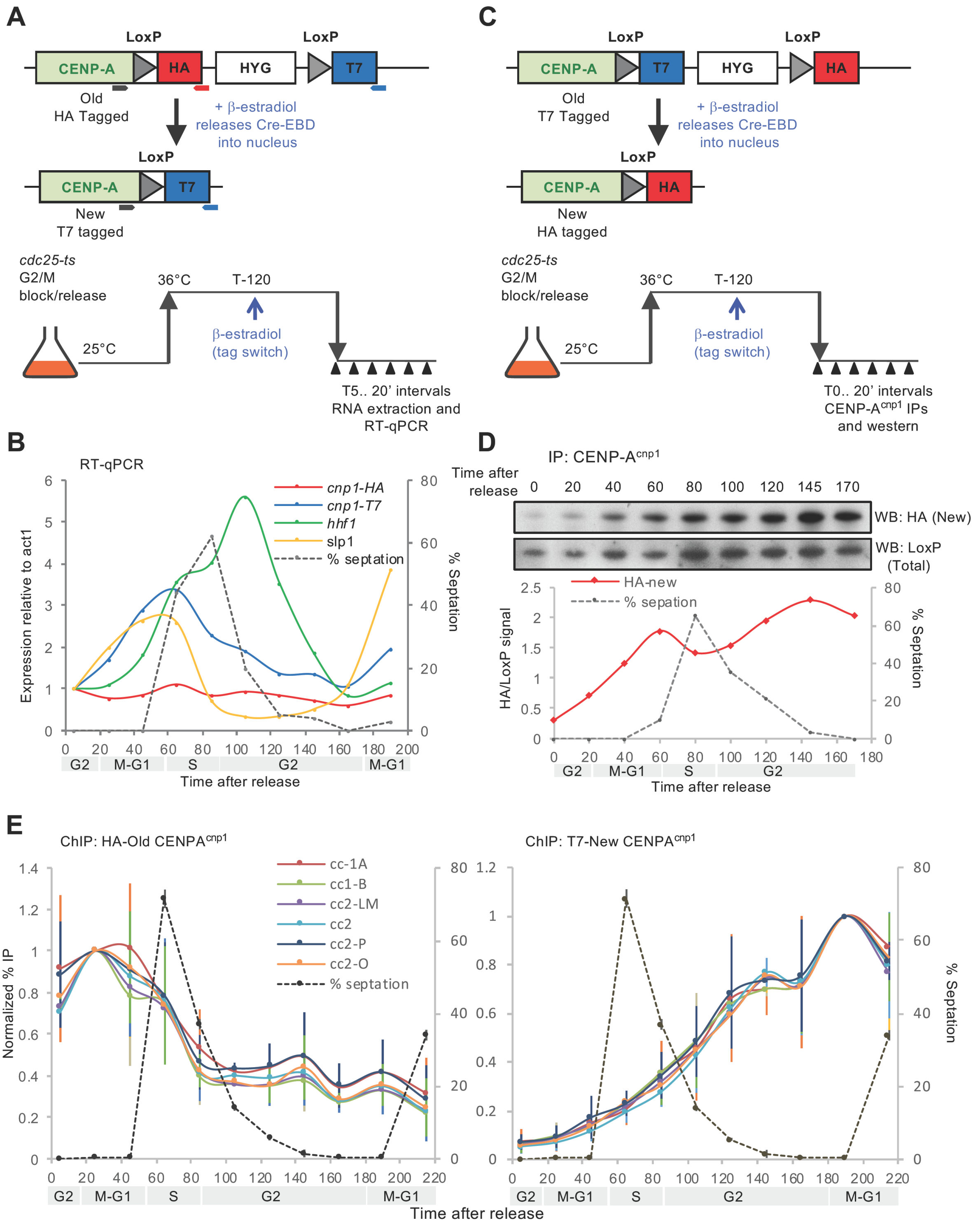

Figure S2. New CENP-A^{Cnp1} is expressed prior to its deposition at centromeres in G2. Related to Figure 1.

(A) Diagram of <u>R</u>ecombination <u>I</u>nduced <u>T</u>ag <u>E</u>xchange (RITE) system and CENP-A^{Cnp1}-RITE tag swap. *cdc25-22* ts mutant cells were blocked in G2 by incubation at 36°C, tag swap was induced by β-estradiol addition. Cells were released synchronously into the cell cycle by shifting to 25°C. Samples for RNA were collected at indicated time-points (T5-T190). Locations of common *cnp1*$^{+}$, HA and T7 tag specific primers are indicated.

(B) RT-qPCR for expression kinetics of *cnp1-HA* or tag swapped *cnp1-T7* genes through cell cycle. Cell cycle dependent exemplar genes *slp1+* (mitosis) and *hhf1*$^{+}$ (S-phase) are shown. Data were normalized to *act1+* expression levels for corresponding time points. Percentage of septated cells and corresponding cell cycle stages are indicated.

(C) Diagram of Recombination Induced Tag Exchange (RITE) system and CENP-A^{Cnp1}-RITE tag swap. *cdc25-22* ts mutant cells were blocked in G2 by incubation at 36°C, tag swap was induced by β-estradiol addition. Cells were released synchronously into the cell cycle by shifting to 25°C. Samples for CENP-A^{cnp1} immunoprecipitations were collected at indicated time-points (T0-T170). CENP-A^{Cnp1} affinity selection was performed using anti-CENP-A^{Cnp1} serum and immunoprecipitated material was probed with anti-LoxP peptide antibody (for detection of total CENP-A^{cnp1}) and anti-HA peptide antibody (for detection of new CENP-A^{cnp1}).

(D) Western blot showing levels of total CENP-A^{Cnp1} (LoxP) and new CENP-A^{Cnp1} (HA). Densitometry was performed using ImageJ and HA western signals were normalized to total CENP-A^{cnp1} (LoxP western) signal for corresponding time points. % septation and cell cycle phases are indicated.

(E) ChIP-qPCR analysis showing the profiles for HA tagged old (left panel) and T7 tagged new (right panel) CENP-A^{Cnp1} during cell cycle at various locations on central core 1 and 2 (related to figure 1B). %IP values were normalized with time point 'T25' values for HA ChIPs and time point 'T190' values for T7 ChIPs respectively. Data shown here are average values from two independent experiments. Error bars represent mean ± SD. Percentage of septated cells is shown to follow the progress of the cell cycle.

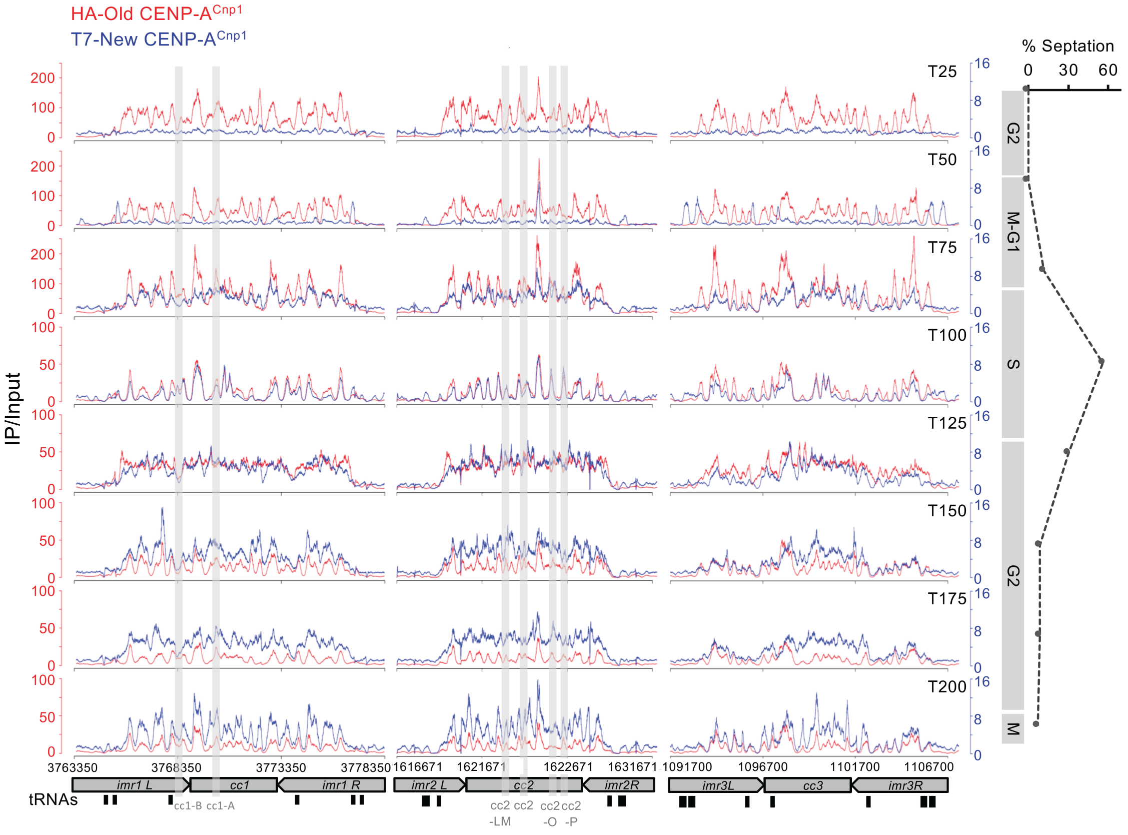

Figure S3. New and old CENP-A^{Cnp1} profiles at fission yeast centromeres through the cell-cycle. Related to figure 2.

ChIP-Seq profiles for HA tagged old (in red) and T7 tagged new (in blue) CENP-A^{Cnp1} through the cell cycle at central core 1 (left panel), central core 2 (middle panel) and central core 3 (right panel). Experimental scheme is same as described in Figure 1A. Respective fold enrichment values (IP relative to Input) for HA (red) and T7 (blue) are shown on Y axes. Corresponding time points, approximate cell cycle phase and chromosomal locations are indicated. Primer locations used in qChIP experiments (Figure 1B and S2E) are indicated by grey bars.

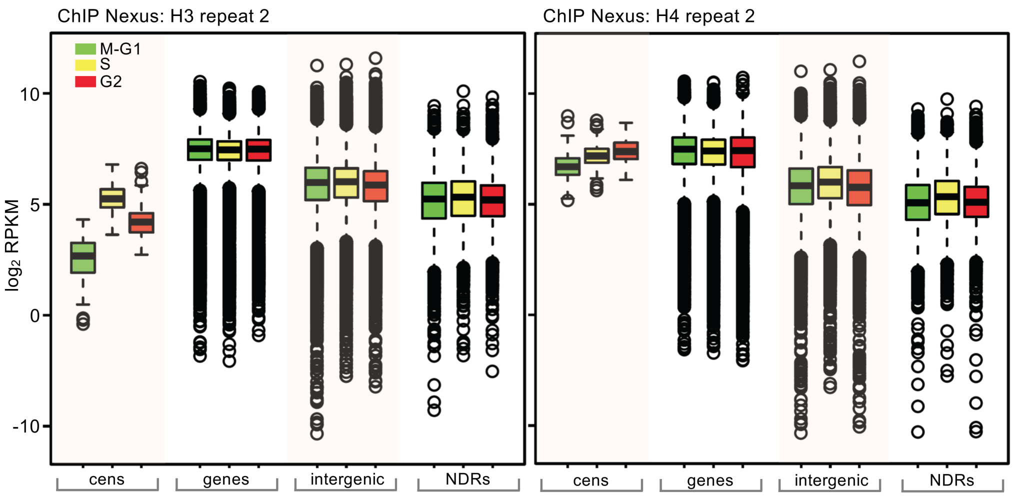

Figure S4. Histone H3 levels increase at centromeres during S-phase. Related to figure 4.

Quantitation of H3 and H4 occupancy by ChIP-Nexus in representative mitosis/G1, S and G2 cell cycle phase samples (replicate 2). Box plot of H3 (left panel) and H4 occupancy levels (right panel) over central core DNA (*cc1*, *cc2*, *cc3* together), gene bodies, intergenic regions and NDRs. Y axis shows $\log_2$ RPKM values for corresponding genomic locations.

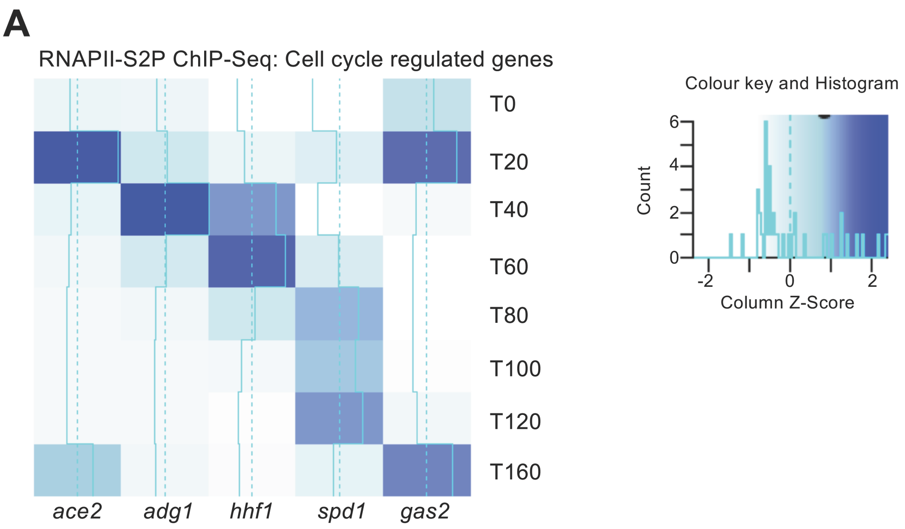

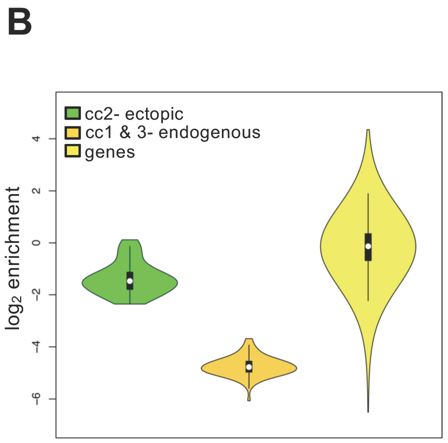

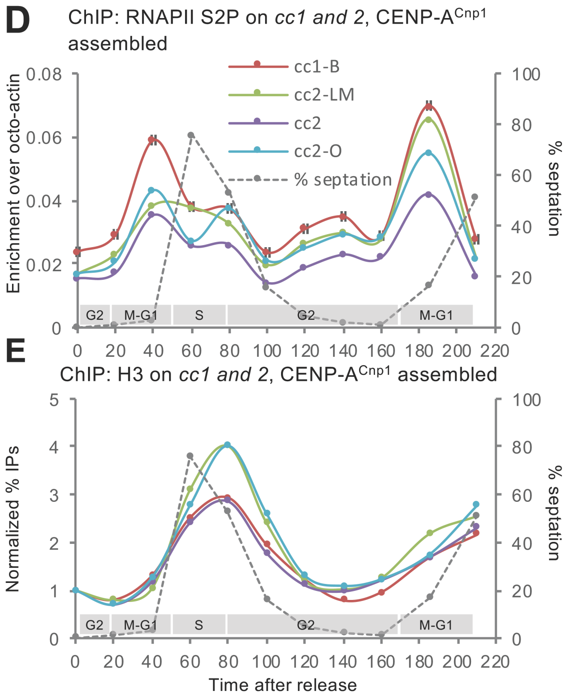

C

RNAPII S2P enrichment	cc1&3	cc2-ectopic	Genes
Maximum	0.07779864	1.08275919	20.3975687
Top 25%	0.04260005	0.45739452	1.27270918
Median	0.03658789	0.36088723	0.91234273
Top 75%	0.03197079	0.28794616	0.62386661
Minimum	0.01491206	0.19569142	0.010989

Figure S5. RNAPII accumulates on centromere DNA in a cell cycle dependent manner. Related to figure 6.

(A) Heat map showing RNAP II S2P enrichment at cell cycle regulated example genes. Enrichments of RNAP II S2P at *ace2*$^{+}$ (M), *adg1*$^{+}$ (G1), *hhf1*$^{+}$ (S), *spd1*$^{+}$ (G2) and *gas2*$^{+}$ (late G2/M) genes are shown corresponding to indicated time points from the ChIP-Seq experiment shown in Figure 6B.

(B) Violin plots of RNAPII-S2P levels over non-centromeric *ura4:cc2* (green) centromeric cc1/cc3 (orange) and genes (yellow) for a representative T0 (G2) sample. Y axis shows log_2 RPKM values for corresponding genomic locations.

(C) Table showing maximum, top 25%, median, top 75% and minimum values for RNAPII-S2P enrichments data shown in (B)

(D) Representative qChIP for RNAPII-S2P levels at endogenous centromeric locations in *cdc25-22* synchronized cell cultures. Y axis: %IP levels were normalized using ChIP levels at *S. octosporus act1*$^+$ from spiked-in chromatin. Septation index and cell cycle phases as indicated.

(E) qChIP for H3 levels from the same cell population as (D). Y axis: %IP levels were normalized first using ChIP levels at *S. octosporus act1*$^+$ from spiked-in chromatin and then to T0 values for each series of samples.

Table S1

List of strains used in the study

Strain no.	Genotype	Reference
FY 1645	*h^+ ade6-210 arg3-D4 his3-D1 leu1-32 ura4-D18*	Lab stock
FY 2352	*h^+ cdc25-22 ade6-210 leu1-32 ura4-D18*	Lab stock
FY A9169	*h^+ ade6-210 cdc25-22 leu1-32 ura4-D18 cnp1:cnp1-loxP-HA-HYG-loxP-T7 ars1:pRAD11-Cre-EBD-leu2+*	This study
FY B2824	*ade6-210 cdc25-22 leu1-32 ura4-D18 cnp1:cnp1-loxP-HA-HYG-loxP-T7 ars1:pRAD13-Cre-EBD-leu2+*	This study
FY B2827	*ade6-210 cdc25-22 leu1-32 ura4-D18 cnp1:cnp1-loxP-T7-HYG-loxP-HA ars1:pRAD13-Cre-EBD-leu2+*	This study
FY B1390	*h^+ kanMX6-Pcnp1-mEGFP-cnp1 ade6-M210 leu1-32 ura4-D18 cdc25-22*	This study
FY A9210	*H3.2-lox-T7-HYG-lox-HA ars1:pRAD11-Cre-EBD-leu2+ cdc25-22 ura4D18/DSE leu1-32*	This study
FY B0324	*cc2Δ6Kb:cc1 Ura4-DSE-sup3e-cc2-ura4+ H3.2-lox-HA-HYG-loxT7 ars1:pRAD11-Cre-EBD-leu2+ cdc25-22 ade6-704-NAT ura4D18/DSE leu1-32*	This study
FY 6443	*h^+ GFP-hht2 leu1-32 ade6-210*	MB1346. Gift from M. Balasubramanian
FY A8240	*h^- kanMX6-Pcnp1-mEGFP-cnp1 ade6-M210 leu1-32 ura4-D18*	JW2595. Gift from Jian-qiu Wu.
FY B3498	*cdc25-22 ade6-210 arg3-D4 leu1-32 ura4-D18 cc1 : arg3*	This study

Table S2

List of primers used in the study

q-cc2-fw	AAACAAACAACGGCACACTG
q-cc2-rev	AAGCCAGCAAATTCCTTGAGT
q-cc2-LM-fw	GCATCTATTGTACTCTCTC
q-cc2-LM-rev	GAAGGATGGATATGCACGT
q-cc2-O-fw	GACTATAACTAGACCACTCAG
q-cc2-O-rev	CTAGATGAATACTCAAGAAAGC
q-cc2-P-fw	CTGCATATTCGACATCTTGAG
q-cc2-P-rev	AGCCTGTCCATCGCAAAAGG
q-cc1-fw	CAGACAATCGTTTCCATTCAG
q-cc1-rev	AGGTGAAGCGTAAGTGAGTG
q-cc1A-fw	GCTAACGAGGCTAACCCACT
q-cc1A-rev	GAGGTTTTTCGTTCTTAGGGCT
q-cc1B-fw	CCGTTGCAACTTACATCAGCA
q-cc1B-rev	CCGGTCGCCAAATAGCAATG
q-act1-fw	GGTTTCGCTGGAGATGATG
q-act1-rev	ATACCACGCTTGCTTTGAG
q-Sp-act1-fw	AGAAGAAATCGCAGCGTTGG
q-Sp-act1-rev	GGTCTACCGACAATCGAGGG
q-So-act1-fw	CAGGTCACCACAGCCTTACA
q-So-act1-rev	CTTGCACATACCAGAGCCGT
q-dg-fw	AATTGTGGTGGTGTGGTAATA
q-dg-rev	GGGTTCATCGTTTCCATTCAG
q-pyk1-5'fw	CTGTCAACCGTCGTACCTCC
q-pyk1-5'rev	TCATACCAGCATCACGGAGC
q-pyk1-GBfw	GTCCGTGTCAACAACAACGG
q-pyk1-GBrev	CTTGACACCGAAGCGGAGAT
q-spd1-5'fw	AGTAGCAAACGCTCCCACAA
q-spd1-5'rev	GGTCATAACTCGCTTGCTGC
q-spd1-GBfw	CTGCCTATAACCCACCGCTT
q-spd1-GBrev	TGCTCGAATGGACGCTTAGT
q-sua1-fw1	TGCTCATTCACCCTGTCGTC
q-sua1-rev1	GGCGGAACCCTTAGGATAGC
q-sua1-fw2	CAGCCTTGAACGAGGATGGT
q-sua1-rev2	AGAAACAACAACGGCAGCAC
q-rpb1-fw	GAAATAGCGGGACAGCCTCA
q-rpb1-rev	TTGCCACGTAATCGACCCTC
q-arg3-P1fw	TGTCGTCTCAGAATGTCGCC
q-arg3-P1rev	GGCAACTGACAGCACTTTCG
q-arg3-P2fw	GCTAAGCCCAAGGACGTCAA
q-arg3-P2rev	TAACAGCAACCTTGGGGTCG
q-arg3-P3fw	TTGTTTACCCCGTCATCCCG
q-arg3-P3rev	TCCATTTGCGGTTCTCTGCT
