# Supplementary material for: Centromere DNA Destabilizes H3 Nucleosomes to Promote CENP-A Deposition during the Cell Cycle
Source: Curr Biol. 2018 Dec 17;28(24):3924–3936.e4. doi: 10.1016/j.cub.2018.10.049 (PMC6303189; doi:10.1016/j.cub.2018.10.049)
Supplement: Document S2. Article plus Supplemental Information [file mmc2.pdf]

Current Biology

Article

Centromere DNA Destabilizes H3 Nucleosomes to Promote CENP-A Deposition during the Cell Cycle

Highlights

- Histone H3 levels increase at centromeres during S phase
- New CENP-A replaces H3 during G2
- Centromere DNA drives H3 dynamics independent of centromere proteins
- Eviction of H3 is coincident with recruitment of elongating RNAPII

Authors

Manu Shukla, Pin Tong,
Sharon A. White, ..., Sandra Catania,
Alison L. Pidoux, Robin C. Allshire

Correspondence

manu.shukla@ed.ac.uk (M.S.),
robin.allshire@ed.ac.uk (R.C.A.)

In Brief

Shukla et al. show that histone H3 is transiently deposited as a placeholder in centromeric chromatin during S phase and subsequently replaced by new centromere-specific CENP-A^{Cnp1} during G2. Eviction of H3 coincides with RNAPII recruitment, suggesting that transcription-coupled events may be involved.

Shukla et al., 2018, Current Biology *28*, 3924–3936
December 17, 2018 © 2018 The Authors. Published by Elsevier Ltd.
https://doi.org/10.1016/j.cub.2018.10.049

CellPress

Current Biology
Article

Centromere DNA Destabilizes H3 Nucleosomes to Promote CENP-A Deposition during the Cell Cycle

Manu Shukla,[1,*] Pin Tong,[1] Sharon A. White,[1] Puneet P. Singh,[1] Angus M. Reid,[1,2] Sandra Catania,[1,3] Alison L. Pidoux,[1] and Robin C. Allshire[1,4,*]
[1]Wellcome Centre for Cell Biology and Institute of Cell Biology, School of Biological Sciences, The University of Edinburgh, Edinburgh EH9 3BF, UK
[2]Present address: Institute of Genetic and Molecular Medicine, Western General Hospital, The University of Edinburgh, Edinburgh EH4 2XU, UK
[3]Present address: Department of Biochemistry and Biophysics, University of California, San Francisco, San Francisco, CA 94158, USA
[4]Lead Contact
*Correspondence: manu.shukla@ed.ac.uk (M.S.), robin.allshire@ed.ac.uk (R.C.A.)
https://doi.org/10.1016/j.cub.2018.10.049

SUMMARY

Active centromeres are defined by the presence of nucleosomes containing CENP-A, a histone H3 variant, which alone is sufficient to direct kinetochore assembly. Once assembled at a location, CENP-A chromatin and kinetochores are maintained at that location through a positive feedback loop where kinetochore proteins recruited by CENP-A promote deposition of new CENP-A following replication. Although CENP-A chromatin itself is a heritable entity, it is normally associated with specific sequences. Intrinsic properties of centromeric DNA may favor the assembly of CENP-A rather than H3 nucleosomes. Here we investigate histone dynamics on centromere DNA. We show that during S phase, histone H3 is deposited as a placeholder at fission yeast centromeres and is subsequently evicted in G2, when we detect deposition of the majority of new CENP-A^{Cnp1}. We also find that centromere DNA has an innate property of driving high rates of turnover of H3-containing nucleosomes, resulting in low nucleosome occupancy. When placed at an ectopic chromosomal location in the absence of any CENP-A^{Cnp1} assembly, centromere DNA appears to retain its ability to impose S phase deposition and G2 eviction of H3, suggesting that features within centromere DNA program H3 dynamics. Because RNA polymerase II (RNAPII) occupancy on this centromere DNA coincides with H3 eviction in G2, we propose a model in which RNAPII-coupled chromatin remodeling promotes replacement of H3 with CENP-A^{Cnp1} nucleosomes.

INTRODUCTION

Centromeres are the defined locations on chromosomes where kinetochores are assembled and that ensure accurate chromosome segregation. In many species, centromere chromatin is distinguished by the presence of CENP-A (also known as cenH3; CID in *Drosophila*, Cse4 in *Saccharomyces*, and Cnp1 in *Schizosaccharomyces*), a histone H3 variant, which substitutes for canonical H3 in specialized nucleosomes that form the foundation for kinetochore assembly [1]. The point centromeres of budding yeast (*Saccharomyces cerevisiae*) are unusual in that they are entirely DNA sequence dependent, because sequence-specific DNA-binding proteins direct CENP-A^{Cse4} and kinetochore assembly [2]. In contrast, regional eukaryotic centromeres (human, fruit fly, plant, fission yeast) assemble CENP-A nucleosomes across extensive DNA regions that are often repetitive [3].

CENP-A is critical in defining where kinetochores are assembled, because its artificial recruitment to non-centromeric chromosomal locations is sufficient to mediate kinetochore assembly [4–6]. Centromere position is normally stable; however, deletion of a normal centromere can allow neocentromere formation at unusual chromosomal locations [7–9]. Moreover, dicentric chromosomes with two centromeres can be stabilized by the inactivation of one centromere without DNA loss [10, 11]. Such observations indicate that CENP-A incorporation and thus centromere positioning exhibits epigenetic plasticity [12, 13].

Overexpression of CENP-A allows its incorporation at novel locations, but the low frequency of kinetochore assembly suggests that several CENP-A nucleosomes may be required [14, 15]. Despite the flexibility associated with CENP-A and thus centromere location, neocentromeres are rare and centromeres usually remain associated with specific DNA sequences [3, 7, 8, 16, 17]. However, despite the conservation of CENP-A and many kinetochore proteins, underlying centromeric DNA is highly divergent [18]. Nevertheless, these centromere sequences allow *de novo* CENP-A and kinetochore assembly following their introduction as naked DNA into cells [19, 20]. Such analyses indicate that centromere DNA is a preferred substrate for CENP-A assembly. The CENP-B DNA-binding protein somehow designates mammalian satellite repeats for CENP-A assembly. However, the mechanisms that promote assembly of CENP-A rather than H3 nucleosomes remain largely unknown [20].

During replication, parental nucleosomes are distributed to both sister chromatids, and new nucleosomes assemble in the resulting gaps by a replication-coupled process. Consequently, half of the histones in nucleosomes on G2 chromatids represent

 © 2018 The Authors. Published by Elsevier Ltd.
This is an open access article under the CC BY license (http://creativecommons.org/licenses/by/4.0/).

“old,” pre-existing subunits, whereas the other half are newly synthesized histones incorporated during replication [21]. Measurements at vertebrate and *Drosophila* centromeres indicate that CENP-A levels are reduced by half during replication [22, 23]. Thus, CENP-A must be replenished each cell cycle outside S phase. Various analyses reveal that in contrast to canonical H3, new CENP-A is incorporated in a replication-independent process confined to a specific portion of the cell cycle. The timing of CENP-A incorporation varies between organisms, cell types, and developmental stages. In mammalian cultured cells and *Drosophila* somatic tissues, new CENP-A is deposited at centromeres in late telophase/early G1 [24, 25]. However, new CENP-A^{CID} is incorporated at *Drosophila* centromeres in cultured cells at metaphase and during anaphase in early embryos [23, 26], whereas it is loaded during G2 in plant tissues [27].

Such studies reveal that some cell types initiate chromosome segregation with a full complement of CENP-A at centromeres, whereas others carry only half the maximal amount and replenish CENP-A levels only after mitotic entry, between metaphase and G1. Nevertheless, the key shared feature is that new CENP-A incorporation is temporally separated from bulk H3 chromatin assembly during S phase. From S phase until the time of new CENP-A deposition, placeholder H3 nucleosomes might be temporarily assembled in place of CENP-A, or gaps completely devoid of nucleosomes may be generated at centromeres [3, 28, 29]. Analysis of human centromere chromatin fibers suggested that H3.3 is deposited as a placeholder in S phase that is later replaced by new CENP-A [30]. However, such repetitive centromeres lack specific sequence landmarks, making precise interpretation difficult, while the cell-cycle dynamics of H3 relative to CENP-A have not been explored in substantial detail at other more tractable regional centromeres. Moreover, cell-cycle-specific replacement of H3 with CENP-A nucleosomes may be directly associated with HJURP/Mis18-mediated CENP-A deposition [31–33]. Alternatively, processes such as transcription, known to induce histone exchange [34], might aid CENP-A deposition by facilitating H3 eviction prior to or coincident with CENP-A deposition. Indeed, transcription has been observed at centromeres and is implicated in CENP-A deposition in several systems [35–45].

Once established, CENP-A chromatin has an innate ability to self-propagate through multiple cell divisions. Such persistence is ensured by associated factors that recognize pre-existing CENP-A nucleosomes and mediate assembly of new CENP-A particles nearby [46–48]. However, the features that distinguish normal centromere DNA as being the preferred location for *de novo* CENP-A chromatin assembly remain unknown, although DNA-binding factors such as CENP-B appear to be involved [20].

Fission yeast, *Schizosaccharomyces pombe*, centromeres are regional and have a distinct experimental advantage in that CENP-A^{Cnp1} nucleosomes and kinetochores are assembled over specific central domains of ~10 kb that are flanked by H3K9me heterochromatin repeats [49, 50]. The unique central CENP-A domain of *cen2* allows detailed analyses unhampered by problematic repetitive centromere DNA [19, 51]. Initial microscopic and genetic analyses indicated that cell-cycle loading of fluorescently tagged CENP-A at the *S. pombe* centromere cluster is either biphasic, occurring both in S phase and G2 [52], or mid-late G2 [53]. However, the dynamics of CENP-A, H3, and RNA polymerase (RNAP)II association have not been examined throughout the cell cycle at an individual specific centromere sequence in any system.

Here we demonstrate that histone H3 is incorporated at *S. pombe* centromeres during S phase, where it serves as an interim placeholder prior to its replacement by new CENP-A during G2. This cell-cycle-regulated program occurs independent of CENP-A and kinetochore assembly, because H3 exhibits similar cell-cycle dynamics on ectopically located centromere DNA, devoid of CENP-A. Moreover, ectopic centromere DNA exhibits intrinsically low H3 nucleosome occupancy and rapid nucleosome turnover. Thus, H3 nucleosomes assembled on centromere DNA are intrinsically unstable. Elongating RNAPII transiently accumulates on this centromere DNA during G2, coincident with the time of H3 eviction. We propose that centromeric DNA drives a program of cell-cycle-coupled events ensuring the sequential and temporally regulated deposition of H3, followed by its replacement with CENP-A resulting in its replenishment. Similar RNAPII transcription-coupled events may contribute to the replacement of H3 with CENP-A on centromeric DNA in other eukaryotes.

RESULTS

New CENP-A^{Cnp1} Is Deposited at Centromeres during G2

Previous single-cell analyses indicated that CENP-A^{Cnp1} levels at *S. pombe* centromeres decline during replication and are replenished during G2 [53], whereas genetic analyses suggested that incorporation occurs during both S and G2 phases [52]. *S. pombe* CENP-A^{Cnp1} transcript and protein levels increase prior to replication, in advance of general histone gene induction (Figures S2A and S2B) [54]. To accurately distinguish between newly synthesized and pre-existing old CENP-A^{Cnp1} protein, we used recombination-induced tag exchange (RITE [55]). All pre-existing “old” CENP-A^{Cnp1} was tagged with the hemagglutinin (HA) epitope. Following β-estradiol-induced nuclear import of Cre-EBD in *cdc25-22*/G2-arrested cells, recombination between Lox sites resulted in “new” T7 epitope-tagged CENP-A^{Cnp1} being expressed in the following G1/S (Figures 1A and S1). After release from G2 (36°C → 25°C shift), the majority of the cell population underwent synchronous cell division as indicated by a peak in septated cell frequency (cytokinesis; 71%) after ~65 min (Figure 1B). G1 is very short in *S. pombe* and S phase coincides with cytokinesis, which is followed immediately by the next G2 [56].

Quantitative chromatin immunoprecipitation (qChIP) analyses at several positions across the central domains (*cc1*, *cc2*) at centromeres 1 and 2 (*cen1*, *cen2*) revealed a drop in old CENP-A^{Cnp1}-HA levels during S phase, consistent with its dilution by distribution to both sister centromeres (Figures 1A, 1B, and S2E). Association of new CENP-A^{Cnp1}-T7 with *cc1*/*cc2* rose to a maximum during the subsequent G2 (time point [T] 190), indicating that most new CENP-A^{Cnp1} is incorporated during G2 (Figures 1B and S2E). Old CENP-A^{Cnp1}-HA and new CENP-A^{Cnp1}-T7 then decline as cells enter the next S phase (T215) as both are distributed to new chromatids. Microscopic analyses showed that following release from the *cdc25-22*/G2 block, old CENP-A^{Cnp1}-HA was detectable at centromeres throughout the time course. In contrast, and in agreement with qChIP, new CENP-A^{Cnp1}-T7 centromere localization was only detected during the next G2 (T100; Figures 1C and 1D).

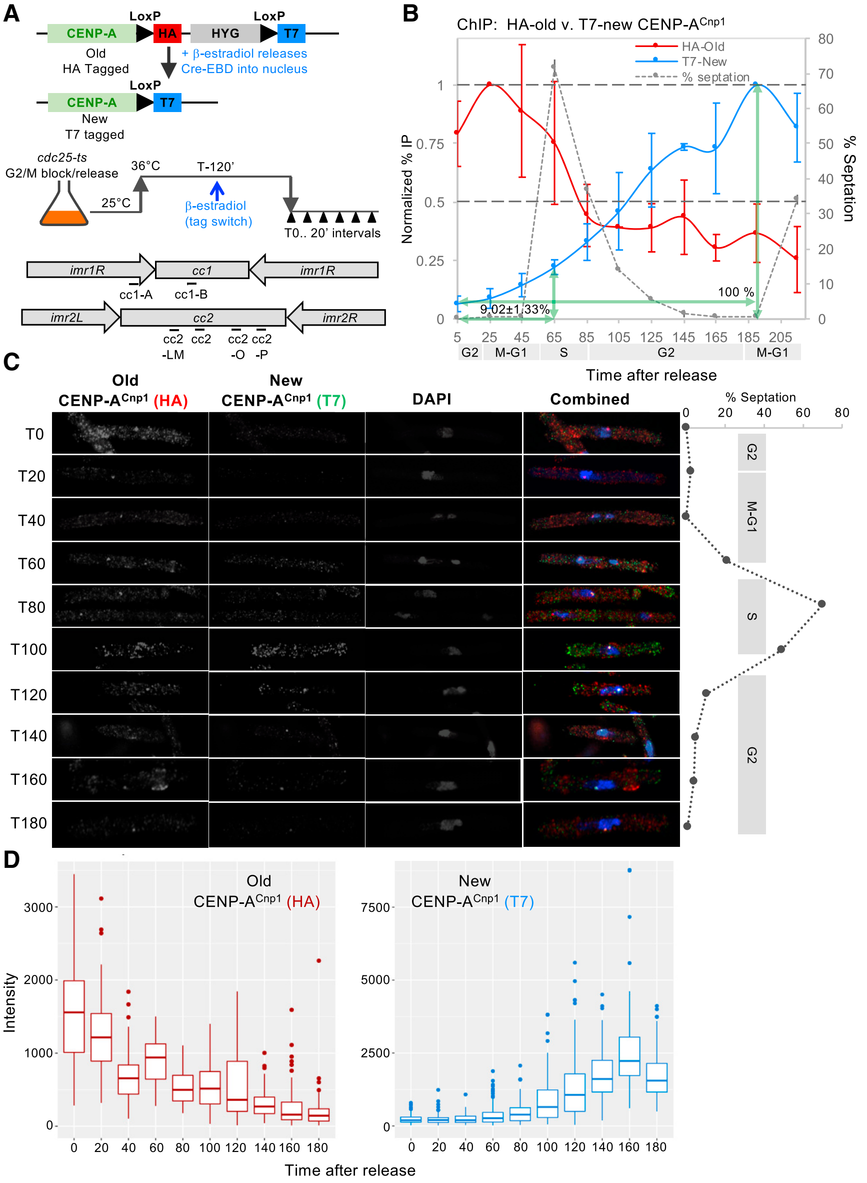

Figure 1. New CENP-A^{Cnp1} Is Deposited at Centromeres during G2

(A) Diagram of the recombination-induced tag exchange (RITE) system and CENP-A^{Cnp1}-RITE tag swap. *cdc25-22* ts mutant cells were blocked in G2 by incubation at 36°C, and tag swap was induced by β-estradiol addition. Cells were released synchronously into the cell cycle by shifting to 25°C. Samples were collected at the indicated time points (T5–T215). Locations of primers used in the qChIP experiment are indicated.

(B) qChIP analysis showing the profiles for HA-tagged old and T7-tagged new CENP-A^{Cnp1} during the cell cycle at *cc2*. y axis: % IP values were averaged over 6 primer pairs (Figures 1A and S2E) and normalized to values at T25 for CENP-A^{Cnp1}-HA and T190 for CENP-A^{Cnp1}-T7. Error bars indicate mean ± SD (n = 2). % septation and cell-cycle stages are as indicated. % area under the curve until T65 relative to T185 is indicated.

(C) Immunolocalization to assess the timing of new CENP-A^{Cnp1}-T7 deposition. Representative images from each time point are shown. The septation index and cell-cycle stages are as indicated.

(D) Quantitation of old CENP-A^{Cnp1}-HA and new CENP-A^{Cnp1}-T7 intensities in individual cells (from C); n = 100 for all time points except T80 (n = 94). Horizontal bars indicate median values ± SD; outliers are shown.

See also Figure S2.

Thus, both qChIP and microscopic analyses show that pre-existing CENP-A^{Cnp1} declines at *S. pombe* centromeres during replication, after which new CENP-A^{Cnp1} is primarily incorporated in G2. Fission yeast centromeres therefore undergo mitosis with a full complement of CENP-A^{Cnp1} chromatin that is halved during their replication. The net loss of CENP-A^{Cnp1} from sister centromeres may result in an increase in the size or numbers of inter-nucleosomal gaps between CENP-A^{Cnp1} nucleosomes. Alternatively, H3-containing nucleosomes may be assembled as temporary placeholders at centromeres during S phase by replication-coupled mechanisms.

CENP-A Profiles Reveal Widespread Deposition and Distinct States during the Cell Cycle

We next performed ChIP sequencing (ChIP-seq) to qualitatively assess the distribution of old HA- and new T7-tagged CENP-A^{Cnp1} across centromeres and the genome, throughout the cell cycle in *cdc25-22* synchronized cells. Following release from G2 and RITE tag swap, samples were collected every 25 min. At T25, old CENP-A^{Cnp1}-HA was detected across *cc2* in a series of ∼20 peaks with relatively shallow intervening troughs, but no significant new CENP-A^{Cnp1}-T7 was detected within centromeres (Figure 2A). As cells proceeded through replication (peak septation/T100), both old CENP-A^{Cnp1}-HA and new CENP-A^{Cnp1}-T7 peaks within *cc2* appeared more distinct with deeper troughs, suggesting that the positioning of CENP-A^{Cnp1}-containing particles becomes more confined (Figure 2A). Most new CENP-A^{Cnp1}-T7 was deposited in G2, when the distinctive "shark-tooth" S phase pattern (T100) became less prominent (T125). Subsequently, a series of peaks gradually returned with intervening shallow troughs, suggesting restoration of mature CENP-A^{Cnp1} chromatin prior to the next mitosis (T200; Figure 2B). Similar dynamics were observed at all centromeres (Figure S3). These qualitative data are generally consistent with qChIP and microscopic analyses, which detect only low levels of new CENP-A^{Cnp1} at centromeres in S phase, whereas most new CENP-A^{Cnp1} incorporation occurs in G2 (Figure 1).

Unexpectedly, ChIP-seq revealed transient widespread low-level incorporation of new CENP-A^{Cnp1}-T7 across the genome prior to S phase, mainly within gene bodies (T50; Figures 3A and 3B). New genic CENP-A^{Cnp1} deposition was most obvious when

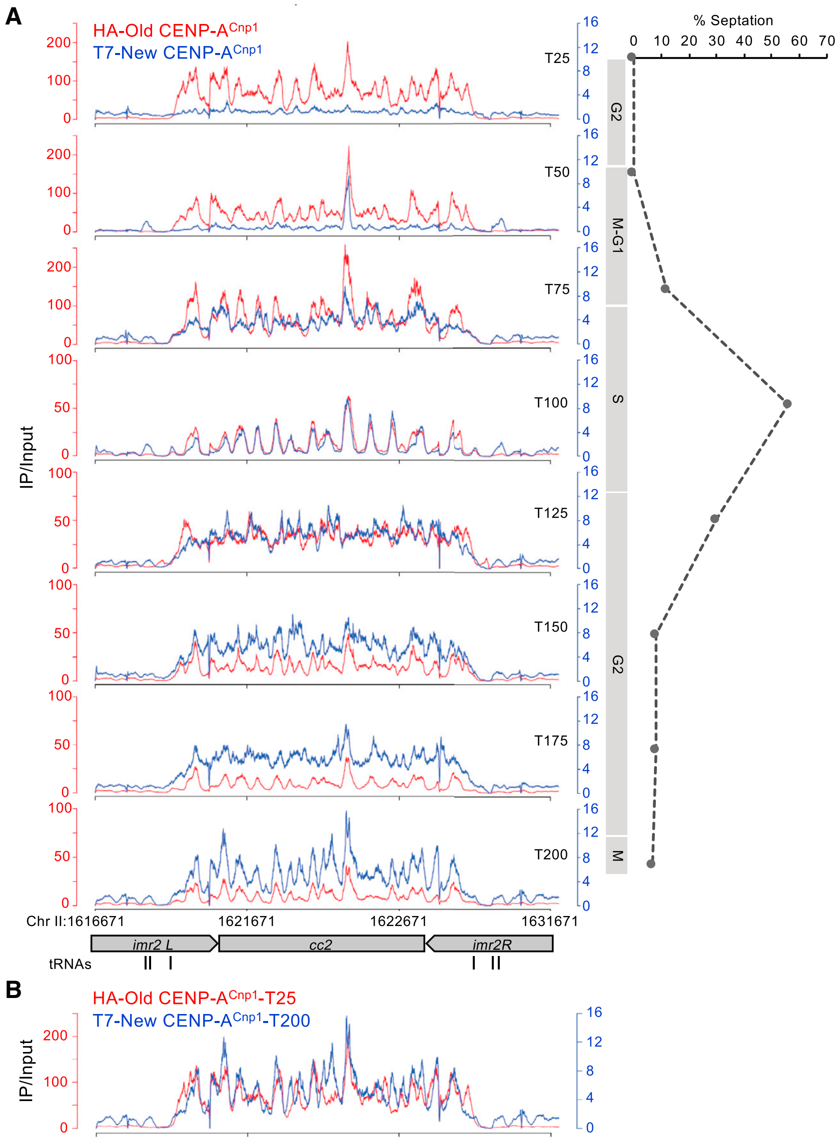

Figure 2. New and Old CENP-A^{Cnp1} Profiles Reveal Distinct States during the Cell Cycle

(A) Cell-cycle ChIP-seq profiles for old CENP-A^{Cnp1}-HA (red) and new CENP-A^{Cnp1}-T7 (blue). Experimental scheme is as in Figure 1A. y axis: respective fold enrichment values corresponding to different time points (IP relative to input) for HA and T7 ChIP are shown. Cell-cycle phases and chromosomal location are as indicated.

(B) Overlay of ChIP-seq profiles for old CENP-A^{Cnp1}-HA at T25 and new CENP-A^{Cnp1}-T7 at T200.

See also Figure S3.

the profile was compared with that of old CENP-A^{Cnp1}-HA over specific genes (Figure 3C). This widespread new CENP-A^{Cnp1} rapidly disappeared as cells enter S phase (T75), and coincided with some new CENP-A^{Cnp1}-T7 accumulation within centromeres (Figure 3A). This non-centromeric signal was exclusive to T50 and did not represent background or an artifact introduced by our analysis. Indeed, in an independent approach, transient incorporation of GFP-CENP-A^{Cnp1} was detected by qChIP prior to two sequential S phases (T20 and T180) within three genes (septation/S phase starts at T60 and T210; Figure 3D).

Histone H3 Is Deposited at Centromeres in S Phase and Evicted during G2

In many eukaryotes, CENP-A incorporation is temporally separated from replication. A placeholder model predicts that H3 should increase at *S. pombe* centromeres during S phase and decline when CENP-A is deposited in G2, whereas H4 levels should remain constant. Conversely, a gap-filling model predicts unaltered H3 occupancy between S and G2 (Figure 4A). To determine the relative cell-cycle dynamics of H3 and CENP-A^{Cnp1}, we analyzed H3, CENP-A^{Cnp1}, and H4 levels at *cc2* in *cdc25-22* synchronized cells by qChIP (Figures 4B–4E). Consistent with a placeholder model, H3 levels over *cc2* increased during S phase (T80), declined throughout G2 (T100–T180), and rose again as cells entered a second S phase (T210; Figure 4C). Reciprocally, centromeric GFP-CENP-A^{Cnp1} levels increased in G2-released cells (T20–T40), declined during S phase (T80), but rose again to maximal levels during G2, coincident with H3 removal (T100–T180). GFP-CENP-A^{Cnp1} levels decreased again as centromeres replicate early in the next S phase (T210; Figure 4D). Importantly, H4 levels, reporting total nucleosome occupancy, remained relatively constant throughout the time course, further suggesting G2-specific H3→CENP-A^{Cnp1} exchange (Figure 4E). Note that variation in synchronization between experiments unavoidably leads to differences in the timing of events between experiments presented here and throughout this study; however, the overall dynamics relative to cell-cycle phases are consistent. Furthermore, comprehensive ChIP-nexus analyses of histones H3 and H4 for representative G1-M, S, and G2 phase samples (Figures 4F and S4) confirmed that H3 association across the central domain of all three centromeres (*cc1*, *cc2*, *cc3*) increases in S phase and subsequently declines in G2. In contrast, H3 association with gene bodies (i.e., open reading frames; ORFs), intergenic regions, and RNAPII promoter-associated nucleosome-depleted regions (NDRs) was unaltered by cell-cycle phase, and H4 levels were similar at centromeres and elsewhere at all cell-cycle phases.

To verify the timing of H3 deposition, we RITE-tagged one (*hht2*$^{+}$/H3.2) of the three genes encoding identical canonical histone H3 proteins. All three *S. pombe* histone H3 proteins (H3.1, H3.2, H3.3) assemble into chromatin during replication; there is no exclusively replication-independent H3 equivalent to the metazoan H3.3 variant [57]. Thus, H3.2-RITE provides a tracer for the dynamics of all H3. In *cdc25-22*/G2-blocked cells all pre-existing old histone H3.2 will be T7 tagged and, following

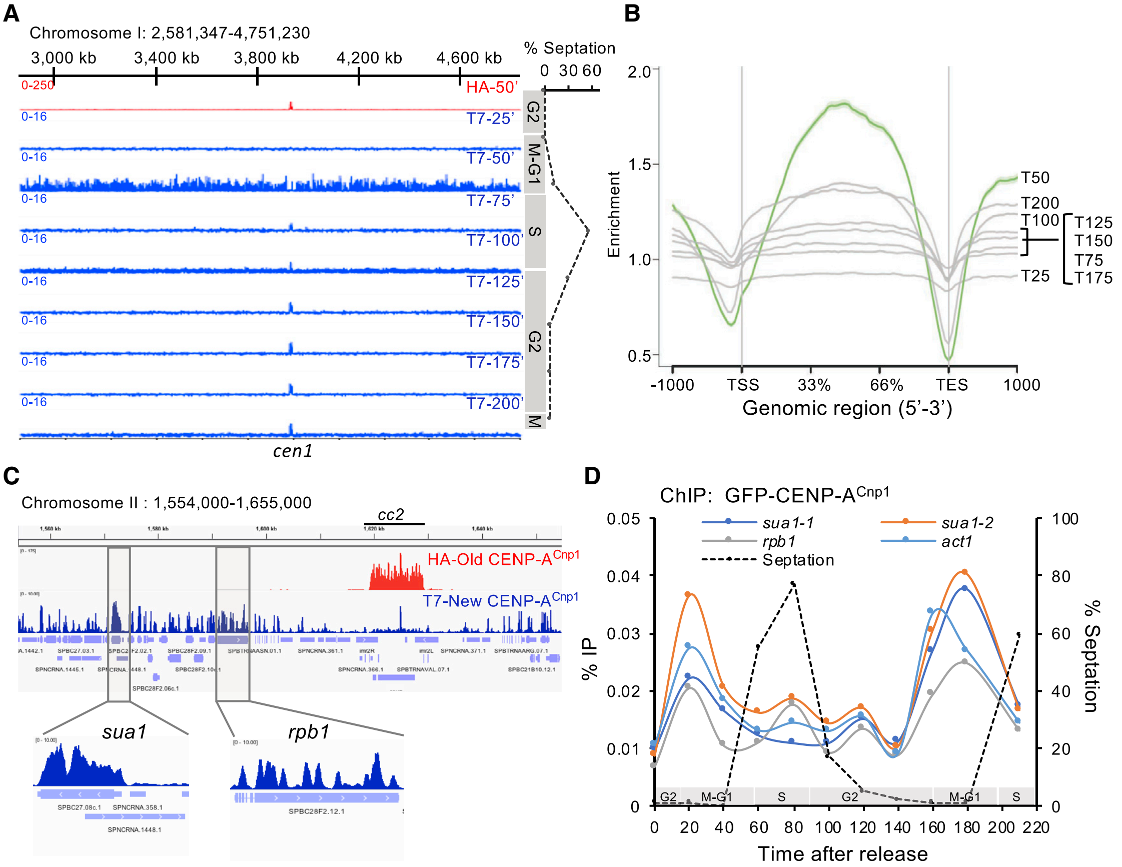

Figure 3. Transient Association of New CENP-A^{Cnp1} throughout Chromosome Arms prior to S Phase

(A) Cell-cycle ChIP-seq profiles of old CENP-A^{Cnp1}-HA (red; T50) and new CENP-A^{Cnp1}-T7 (blue; T25–T200). Fold enrichment (IP/input), % septation, cell-cycle phases, and chromosomal location are as indicated.

(B) Association of new CENP-A^{Cnp1} with genes throughout the cell cycle. ChIP-seq enrichment values for new CENP-A^{Cnp1}-T7 from the indicated time points are shown across average positions within or flanking all genes.

(C) Genome browser view from chromosome II for a T50 sample showing association of old CENP-A^{Cnp1}-HA (red) and new CENP-A^{Cnp1}-T7 (blue). Chromosomal locations of genes and *cen2* are indicated. Bottom: expanded profiles of exemplar *sua1*$^+$ and *rpb1*$^+$ genes showing incorporation of new CENP-A^{Cnp1}-T7.

(D) Representative ChIP for GFP-CENP-A^{Cnp1} cell-cycle incorporation at exemplar genes (*sua1*$^+$, *rpb1*$^+$, and *act1*$^+$). y axis: % IP values. The septation index and cell stages are as indicated.

T7→HA tag swap induction during the G2 block, all new H3.2 will be HA tagged (Figure 4G).

By the end of S phase (T150, after septation peak), qChIP revealed that old H3.2-T7 levels drop within centromeres and on the constitutively expressed *act1*$^+$ gene (Figure 4H). This decline must represent the distribution of parental H3.2-T7 nucleosomes to sister chromatids. New H3.2-HA accumulated within centromeric *cc2* during S/early G2 (T90–T150) but declined during mid-late G2 (Figure 4H). The loss of this new H3.2-HA coincided with the time when most new CENP-A^{Cnp1} deposition was detected within centromeres (Figure 1) and when total centromere-associated GFP-CENP-A^{cnp1} was found to increase (Figure 4D). No such reduction in H3.3-HA levels was observed on the *act1*$^+$ gene during G2; instead, new H3.2-HA was incorporated during replication and remained in place throughout the following G2 (Figure 4H). ChIP for H3 on the same samples confirmed that total H3 increased on centromeric *cc2* during S phase but subsequently declined in G2, whereas little overall cell-cycle change occurred on *act1*$^+$ (Figure 4H).

Together, these analyses show that H3 accumulates within the CENP-A^{Cnp1}-containing regions of centromeres during S phase but is later removed during G2. New CENP-A^{Cnp1} is incorporated within these centromeric regions during G2, coincident with H3 removal. We conclude that H3 nucleosomes assembled within centromeres during S phase serve as temporary placeholders that are replaced by CENP-A^{Cnp1} nucleosomes during G2.

Centromere DNA Alone Drives Histone H3 Cell-Cycle Dynamics

The above dynamics of H3 and CENP-A^{Cnp1} at centromeres may be entirely dictated by kinetochore-associated CENP-A^{Cnp1} loading factors (e.g., HJURP^{Scm3}, Mis18 [33, 58, 59]), or central domain sequences themselves might enforce processes that promote such dynamics. To determine whether centromere DNA itself programs H3 cell-cycle dynamics, we utilized cells carrying 8.5 kb of *cc2* DNA at the non-centromeric *ura4* locus and with endogenous *cen2-cc2* replaced with *cen1* central

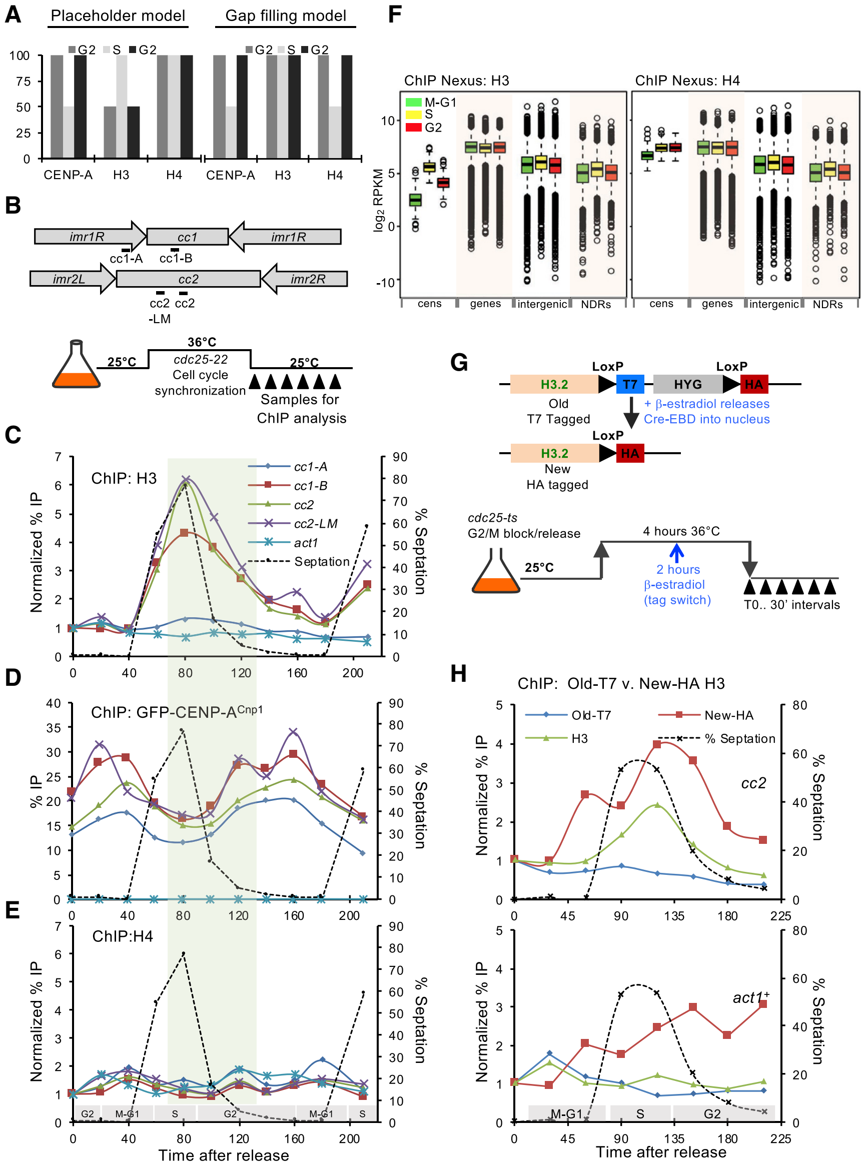

Figure 4. Histone H3 Acts as an Interim Placeholder for CENP-A^{Cnp1} during S Phase

(A) Illustrative graph showing the expected behavior of CENP-A^{Cnp1} and histones H3 and H4 at centromeres as a result of placeholder or gap-filling models.

(B) Schematic of *S. pombe* centromere 1 and 2 organization. Positions of primers used for qChIP are as indicated. A schematic of *cdc25-22* block-release cell-cycle synchronization to assess cell-cycle histone dynamics at centromeres is shown.

(C–E) Representative qChIP experiment measuring histone H3 (C), GFP-CENP-A^{Cnp1} (D), and histone H4 (E) levels at the indicated positions at *cen1*/*cen2* (B), and on *act1$^+$*, in the same samples throughout the cell cycle. For H3 and H4 (C and E), % IP levels were normalized first using ChIP levels at *Schizosaccharomyces octosporus act1$^+$* from spiked-in chromatin and then to T0 values for each series of samples. Green rectangle: time of H3→CENP-A^{Cnp1} exchange.

(F) Quantitation of H3 and H4 occupancy by ChIP-nexus in cell-cycle phases. Boxplot of H3 (left panel) and H4 occupancy (right panel) over central domains (*cc1*, *cc2*, *cc3* together), gene bodies, intergenic regions, and NDRs. y axis: $\log_2$ RPKM (reads per kilo-base per million mapped reads) values.

(G) Diagram of H3.2-RITE T7→HA swap. The RITE cassette was integrated in-frame downstream of *hht2$^+$*. Cell-cycle block release and tag swap induction are as described in Figure 1A.

(H) qChIP for old H3-T7, new H3-HA, and total H3 levels on endogenous *cc2* at *cen2* (top panel) and *act1$^+$* (bottom panel) throughout the cell cycle. y axis: % IP levels were normalized to T0 values for each series of samples.

See also Figure S4.

core DNA (*cc1*) so that ectopic *cc2* (*ura4:cc2*) is the only copy of this element (Figure 5A [37, 38]). qChIP on asynchronous cells confirmed that ectopic *ura4:cc2* was completely devoid of CENP-A^{Cnp1} and assembled in H3 chromatin, albeit at low levels relative to *act1$^+$* (Figure 5B). Remarkably, qChIP on *cdc25-22* synchronized cells revealed that, as at endogenous centromeres (*cc1*), H3 accumulated on ectopic *ura4:cc2* DNA (*cc2*) during S phase (T60–T100) but subsequently decreased during G2 (T90–T150; Figure 5C). H3 levels rose again during the next S phase (T210). We conclude that innate properties of central domain DNA promote H3 nucleosome assembly during S phase and their later removal during G2.

H3 Nucleosomes Exhibit Low Occupancy and High Turnover on Ectopically Located Centromere DNA

The cell-cycle dynamics of H3 gain and loss on ectopic central domain DNA suggest that H3 nucleosomes assembled on these sequences may be inherently unstable. We next compared the steady-state levels of H3 and H4 associated with native cen2-*cc2*, ectopic *ura4:cc2*, and with gene bodies, intergenic regions, and promoter-associated NDRs using ChIP-nexus [60]. As expected, only low levels of H3 were detected over the central domains of endogenous centromeres (*cc1*, *cc2*, *cc3*), where most H3 is replaced by CENP-A^{Cnp1} (Figure 5D). In contrast, H4 (a component of H3 and CENP-A^{Cnp1} nucleosomes) levels throughout centromeric central domains and gene bodies were equivalent. Remarkably, significantly lower levels of H3 and H4 were detected at ectopic *ura4:cc2* DNA, similar to those within intergenic regions and NDRs. These analyses suggest that H3 nucleosome assembly is strongly disfavored on ectopic *ura4:cc2*, whereas CENP-A^{Cnp1} nucleosomes exhibit greater stability on *cc2* DNA within a functional centromere.

We next utilized H3.2-RITE to measure H3 turnover on ectopic *ura4:cc2* in comparison to heterochromatic repeats and highly transcribed genes in G2-arrested cells. The H3.2-HA→T7 tag swap was induced in *cdc25-22*/G2-arrested cells

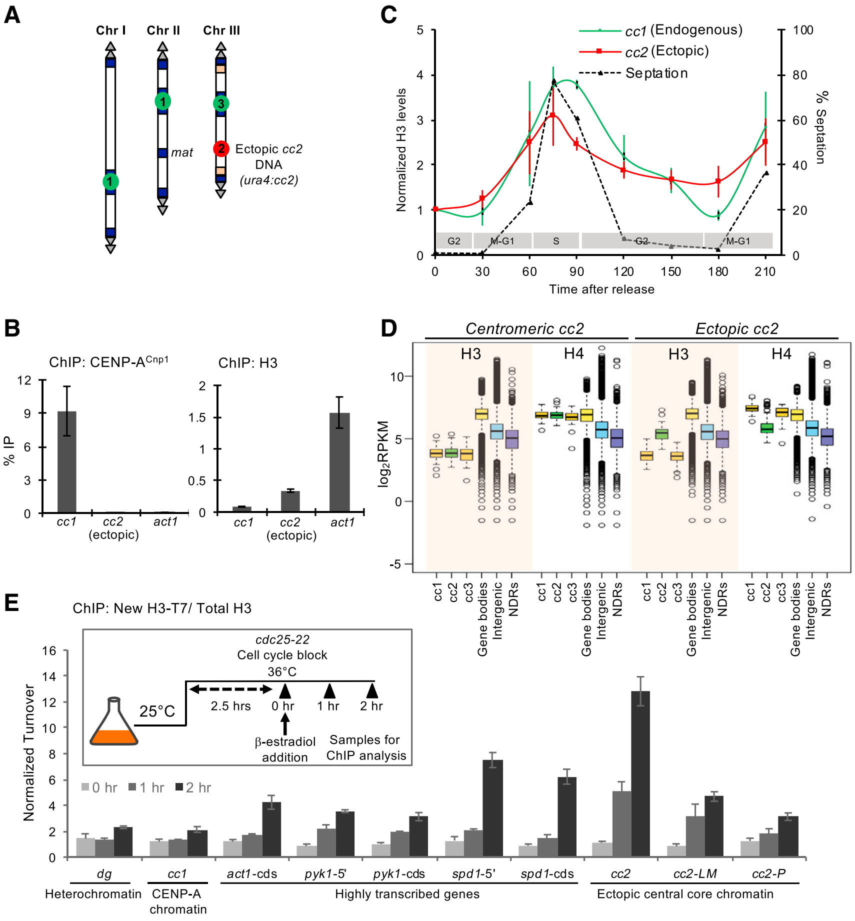

Figure 5. Centromere DNA Destabilizes the H3 Nucleosome and Drives Histone H3 Cell-Cycle Dynamics

(A) Diagram of cells with 8.5 kb of *cc2* DNA from *cen2* inserted at an ectopic non-centromeric locus (*ura4:cc2*) and a 6-kb region of *cen2-cc2* replaced with 5.5 kb of *cen1-cc1* DNA. This allows specific ChIP on unique *ura4:cc2* that lacks CENP-A^{Cnp1}.

(B) qChIP for CENP-A^{Cnp1} and H3 levels at centromere-located *cc1* DNA and ectopic non-centromeric *ura4:cc2* DNA. Control non-centromeric transcribed gene: *act1*$^+$. Error bars indicate mean ± SD (n = 3).

(C) qChIP for H3 levels in *cdc25-22* synchronized cell populations at the indicated time points (Ts) following release into the cell cycle. The septation index and cell-cycle stages are as indicated. y axis: % IP values were normalized to ChIP levels at *act1*$^+$ and then to T0. Error bars indicate mean ± SD (n = 3).

(D) Quantitation of H3 and H4 occupancy by ChIP-nexus. Boxplot of H3 (shaded rectangles) and H4 occupancy over central core DNA (*cc1*, *cc2*, *cc3*), gene bodies, intergenic regions, and NDRs in wild-type cells (centromeric *cc2*) and cells carrying unique non-centromeric *ura4:cc2* (ectopic *cc2*). y axis: $\log_2$ RPKM values.

(E) qChIP for new H3.2-T7 incorporated during *cdc25-22*/G2 arrest into heterochromatin *dg* repeats and *cen1 cc1*, highly transcribed genes (*pyk1*$^+$, *spd1*$^+$ *act1*$^+$), and three locations within non-centromeric ectopic *ura4:cc2*. y axis: normalized turnover represents H3.2-T7 % IP values normalized to the respective total H3 values for each sample and then to the T0 value for one replicate. Error bars indicate mean ± SD (n = 3). Inset: diagram of experimental setup to assess replication-independent H3 turnover. H3.2-RITE HA→T7 tag swap was β-estradiol induced in *cdc25-22*/G2-blocked cells after 2 hr at 36°C, and samples were then collected after 0, 1, and 2 additional hours at 36°C and analyzed by ChIP.

and new histone H3.2-T7 incorporation was monitored (Figure 5E). As expected, only low levels of new H3-T7 were incorporated into heterochromatin, where histone turnover is low [61, 62]. Similarly, the turnover of H3 nucleosomes within *cc1* at endogenous *cen1* was also low, presumably because most H3 nucleosomes were replaced by CENP-A^{Cnp1} during G2. Consistent with transcription-coupled nucleosome exchange, high levels of new H3-T7 were incorporated into chromatin associated with the highly expressed *act1*$^+$, *pyk1*$^+$, and *spd1*$^+$ genes after 1 and 2 hr. Intriguingly, high levels of new H3-T7 were detected on ectopic *ura4:cc2* after just 1 hr, indicating extensive H3 turnover. These data suggest that central domain DNA may render assembled H3 nucleosomes unstable so that they are continually displaced, resulting in low nucleosome occupancy.

RNAPII Accumulates on Centromere DNA Coincident with H3 Removal

Transcription has been implicated in the deposition of CENP-A at centromeres [44, 45], and defective RNAPII elongation is known to enhance CENP-A^{Cnp1} deposition on naive *cc2* DNA in *S. pombe* [37, 38]. Indeed, western analysis indicates that elongating RNAPII-S2P (serine 2 phosphorylated) is present in affinity-selected GFP-CENP-A^{Cnp1} chromatin (Figure 6A). To determine whether transcription, H3 turnover, and CENP-A^{Cnp1} deposition on *cc2* DNA might be coupled, we performed ChIP-seq for elongating RNAPII on synchronized cells carrying ectopic *ura4:cc2*. The cyclical association of RNAPII-S2P with known cell-cycle-regulated genes in synchronized cultures confirmed that we detect the cell-cycle-regulated engagement of RNAPII-S2P (Figure S5A).

In G2-blocked cells (T0), relatively high levels of elongating RNAPII were detected on ectopic *ura4:cc2* (Figures 6B, S5B, and S5C). However, upon release into the cell cycle, associated RNAPII-S2P rapidly declined to a minimum (T40) prior to S phase and accumulated again during G2 (T80–T120; Figure 6C). RNAPII-S2P qChIP at several locations across *ura4:cc2* confirmed that elongating RNAPII occupancy falls in advance of S phase (T40), increases during G2 (T100–T140), and again decreases to a minimum before the next S phase (T160–T180; Figure 6D). Thus, elongating RNAPII reaches maximal levels on ectopic *ura4:cc2* during G2 when H3 is removed (Figure 5C),

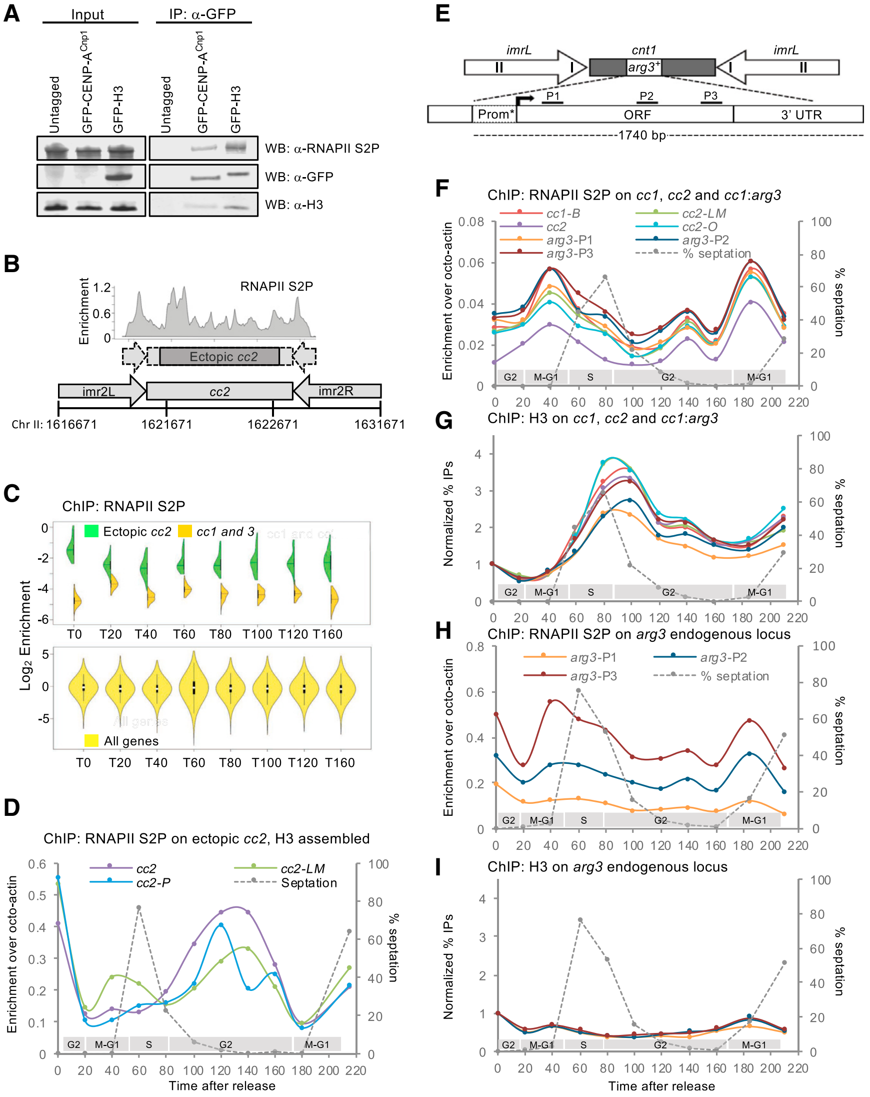

Figure 6. RNAPII Accumulates on Centromere DNA Coincident with H3 Removal

(A) RNAPII-S2P association with native affinity-selected CENP-A^{Cnp1} chromatin. GFP-CENP-A^{Cnp1} or GFP-H3 chromatin was affinity selected from MNase-released chromatin using anti-GFP antibody. Untagged chromatin served as a control. Anti-GFP, anti-H3, and anti-RNAPII-S2P western analyses of inputs and immunoprecipitates are as indicated. WB, western blot.

(B) An RNAPII-S2P ChIP-seq profile over ectopic *ura4:cc2* DNA is shown for a representative G2/T0 sample. y axis: enrichment (IP/input). Chromosome coordinates, a diagram of ectopic *cc2*, and the unique region within ectopic *cc2* are indicated (dark shading).

(C) Violin plots of RNAPII-S2P levels over non-centromeric *ura4:cc2* (green), centromeric *cc1/cc3* (orange), and genes (yellow) in T0–T160 from *cdc25-22* synchronized cell cultures. y axis: $\log_2$ enrichment values.

(D) Representative qChIP for RNAPII-S2P levels on non-centromeric *ura4:cc2* in *cdc25-22* synchronized cell cultures. y axis: % IP levels were normalized using ChIP levels at *S. octosporus act1*$^+$ from spiked-in chromatin. The septation index and cell-cycle phases are as indicated.

(E) Diagram of the *arg3*$^+$ gene inserted at central core 1 (*cc1:arg3*$^+$). qPCR primer locations are indicated.

(F) Representative qChIP for RNAPII-S2P levels on endogenous centromeric locations and *cc1:arg3*$^+$ in *cdc25-22* synchronized cell cultures. Normalizations are as in (D). The septation index and cell-cycle phases are as indicated.

(G) qChIP for H3 levels from the same cell population as (F). y axis: % IP levels were normalized first using ChIP levels at *S. octosporus act1*$^+$ from spiked-in chromatin and then to T0 values for each series of samples.

(H and I) qChIP for RNAPII-S2P (H) and H3 (I) levels on the *arg3*$^+$ endogenous location in *cdc25-22* synchronized cell cultures. Normalizations are as in (D) and (G), respectively. The septation index and cell-cycle phases are as indicated.

See also Figure S5.

suggesting that transcriptional elongation may be involved in H3 removal.

At endogenous CENP-A^{Cnp1}-coated *cc1* and *cc2*, RNAPII-S2P also rises during G2 (T120–T140; Figures 6F and S5D), coincident with loss of H3 (Figures 6G and S5E). The cell-cycle RNAPII-S2P association profile on centromeric *cc1* and *cc2* is confounded by a more dominant RNAPII-S2P peak during mitosis (Figure 6F). The mitotic recruitment of RNAPII appears to be conserved but is likely to be functionally distinct from the RNAPII-S2P that is coupled to CENP-A dynamics [41, 63]. This M phase RNAPII-S2P peak is probably kinetochore imposed, as it was not detected on ectopic *ura4:cc2*. The increase in RNAPII-S2P at endogenous centromeres during G2 is clearly less conspicuous than that detected at ectopic *cc2* (see Discussion). Regardless, RNAPII-S2P recruitment appears to increase at both endogenous centromeres and ectopic *cc2* during G2, when H3 levels decline but CENP-A levels increase.

Marker genes inserted in central domains of *S. pombe* centromeres become assembled in CENP-A^{Cnp1} chromatin [64]. To determine whether marker genes also acquire other central domain properties, RNAPII-S2P and H3 levels associated with a promoter-attenuated *arg3*$^+$ gene inserted in *cc1* of *cen1* (*cc1:arg3*$^+$) were analyzed throughout the cell cycle (Figure 6E [65]). Whereas *arg3*$^+$ at its endogenous location showed a relatively flat H3 cell-cycle profile and an RNAPII peak in M-G1 consistent with its known cell-cycle regulation (Figures 6H and 6I [66]), *cc1:arg3*$^+$ displayed distinctly different H3 and RNAPII-S2P profiles, mirroring that of flanking *cc1* centromeric chromatin (Figures 6F and 6G). Thus, the normal *arg3*$^+$ gene H3 chromatin dynamics appear to be overridden by a program imposed by surrounding central domain chromatin: the unremarkable H3 cell-cycle pattern on *arg3*$^+$ is converted to a "placeholder" pattern, where H3 incorporation rises in S phase and falls dramatically in G2, accompanied by a distinctive RNAPII-S2P profile.

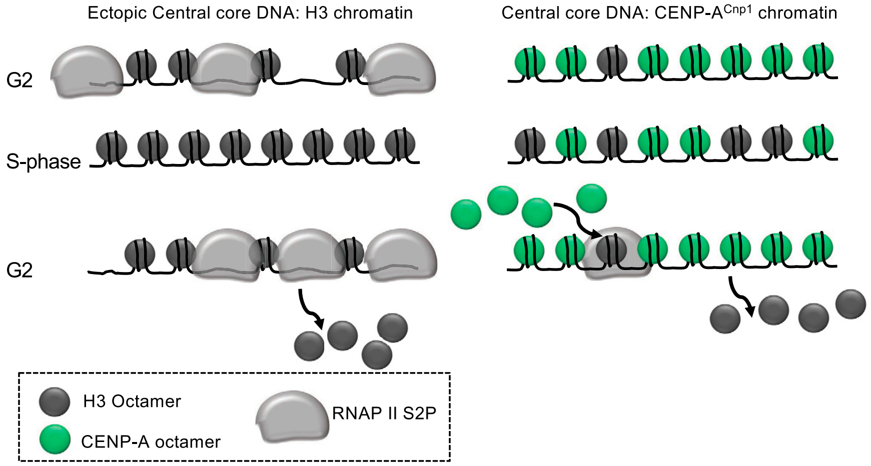

Figure 7. Model for Centromere DNA-Driven Histone Dynamics

Left: ectopic *cc2* centromere DNA drives H3 deposition in S phase and RNAPII recruitment and H3 eviction in G2 despite the absence of CENP-A^{Cnp1} chromatin or CENP-A^{Cnp1} dedicated deposition machinery. Right: new CENP-A^{Cnp1} is incorporated at centromeres in G2. H3 nucleosomes are transiently assembled as placeholders at centromeres during S phase and replaced in the following G2 by new CENP-A^{Cnp1} nucleosomes. RNAPII recruited during G2 facilitates H3 nucleosome disassembly.

Our analysis suggests that central domain DNA from *S. pombe* centromeres may program transcription-coupled H3 nucleosome destabilization during G2, resulting in their replacement with CENP-A^{Cnp1}-containing nucleosomes when a sufficient route of CENP-A^{Cnp1} supply is available.

DISCUSSION

To understand more fully how CENP-A^{Cnp1} chromatin domains are established and propagated on particular sequences across multiple generations, we focused on the cell-cycle dynamics of *S. pombe* centromere-associated chromatin. RITE tag swap experiments allowed us to determine that most new CENP-A^{Cnp1} is incorporated at *S. pombe* centromeres during G2. Our analyses also conclusively demonstrate that histone H3 is deposited as a temporary placeholder during S phase. Importantly, these measurements pinpoint a specific window in G2 where H3→CENP-A^{Cnp1} nucleosome exchange occurs. In addition, we show that the CENP-A^{Cnp1} chromatin profile is highly dynamic, exhibiting stage-specific patterns throughout the cell cycle. Strikingly, we find that ectopically located centromere DNA assembles inherently unstable H3 nucleosomes that exhibit high turnover rates, and during replication this DNA also directs elevated incorporation of H3 nucleosomes that are evicted in the following G2 when elongating RNAPII is recruited. Because the ectopic *cc2* insert is 8.5 kb in length, it thus seems unlikely that the observed dynamics are influenced by flanking non-centromeric chromatin. Together, our analyses support a model in which centromere DNA possesses inherent properties that may drive a sequence-directed cell-cycle-regulated program that promotes H3 nucleosome assembly in S phase and subsequent eviction in G2 allowing the incorporation of new CENP-A^{Cnp1} nucleosomes (Figure 7).

The cell-cycle timing of new CENP-A loading at centromeres varies between organisms; however, a conserved feature is that new CENP-A incorporation is uncoupled from replication, when most new H3 chromatin assembly occurs [23–27]. Key components required for CENP-A chromatin maintenance at human centromeres (Mis18 complex, Mis18BP1^{KNL2}, HJURP) are recruited to centromeres in a temporally restricted manner prior to, or coincident with, new CENP-A deposition [5, 67, 68]. However, Mis18 and Scm3^{HJURP} remain associated with *S. pombe* centromeres throughout most of the cell cycle, apart from a brief period in mitosis [33, 58]. Thus, *S. pombe* centromeres might be more generally competent for new CENP-A^{Cnp1} deposition throughout the cell cycle. It was therefore critical to specifically distinguish old parental CENP-A^{Cnp1} from newly synthesized CENP-A^{Cnp1} at individual centromeres. Previous studies have suggested either biphasic (both S and G2) or G2-specific replenishment of *S. pombe* CENP-A^{Cnp1} and relied on microscopic measurement at the centromere cluster rather than association with chromatin [52, 53]. RITE tagging allowed detailed examination of the behavior of both old and new CENP-A^{Cnp1} at specific centromeres. Our data show that in *S. pombe* the majority of the new CENP-A^{Cnp1} deposition occurs during G2, with only low-level new CENP-A^{Cnp1} deposition during replication. These analyses refine previous studies of CENP-A^{Cnp1} replenishment and suggest that other mechanisms, apart from the temporally regulated recruitment of CENP-A loading factors to centromeres, can influence the cell-cycle stage-specific restriction of new CENP-A^{Cnp1} incorporation.

An unexpected finding was that new CENP-A^{Cnp1} is transiently incorporated within genic regions along chromosome arms prior to replication. In *S. pombe*, the gene encoding CENP-A^{Cnp1} (*cnp1*$^{+}$) is known to be expressed prior to the peak of canonical histone gene expression in advance of S phase (Figure S2B [54]). As a consequence of this earlier CENP-A^{Cnp1} expression, there is a brief window during the cell cycle where the ratio of soluble CENP-A^{Cnp1} versus canonical histone H3 may be skewed. This could account for the transient widespread incorporation of CENP-A^{Cnp1} within genes that may be mediated by transcription-coupled nucleosome turnover. In essence, this portion of the cell cycle exhibits similarity to cells overexpressing CENP-A^{Cnp1} [69]. Conversely, histone H3 can replace CENP-A^{Cnp1} within *S. pombe* centromeres when the H3:CENP-A^{Cnp1} ratio is perturbed [64]. The CENP-A^{Cnp1} incorporation that we detect along *S. pombe* chromosome arms is rapidly removed. In other organisms, CENP-A can also be incorporated throughout the genome, with or without overexpression [14, 15, 37, 70]. This “sampling” by genome-wide CENP-A^{Cnp1} incorporation every cell cycle could potentially contribute to the formation of neocentromeres when conditions demand.

Our comparison of the relative levels of CENP-A^{Cnp1}, H3, and H4 within the CENP-A domain of centromeres revealed that

canonical histone H3 is transiently deposited as a placeholder during S phase and later exchanged for new CENP-A^{Cnp1} in G2. At human centromeres, chromatin fiber analysis suggests that the replication-independent H3.3 variant acts as an interim placeholder for CENP-A [30], and thus H3 placeholder function may be conserved across eukaryotes where new CENP-A deposition is separated from replication. The use of H3 as a transient placeholder in *S. pombe* is important, because it identifies a cell-cycle period when specific H3→CENP-A replacement events must take place and involve factors that mediate histone exchange or complete nucleosome turnover.

Where tested, centromere DNA sequences are clearly a preferred substrate for *de novo* CENP-A assembly [51, 71]. The embedded features that identify centromere DNA for efficient CENP-A assembly involve DNA-binding factors such as CENP-B and processes such as transcription [20, 35, 36, 39–45]. Transcription is a potent chromatin remodeling mechanism and is coupled to histone exchange; new histone H3.3 is deposited within transcribed genes and H2A is exchanged for the variant H2A.Z in the highly dynamic NDR promoter-proximal nucleosomes of many organisms [72]. Our finding that elongating RNAPII-S2P increases on S. *pombe* centromere DNA simultaneous with H3 eviction and CENP-A^{Cnp1} incorporation is compatible with a model where transcription-coupled remodeling events define this centromeric DNA by driving H3→CENP-A exchange. Indeed, RNAPII is known to be an excellent nucleosome disassembly and remodeling machine [73, 74]. Consistent with a role of transcription in facilitating CENP-A^{Cnp1} incorporation, there is a high density of transcriptional start sites within centromere DNA that may promote pervasive low-quality transcription [38].

Elongating RNAPII was found to increase at both endogenous centromeres and on ectopically located central domain DNA (*ura4:cc2*) during G2. Ectopic *cc2* DNA lacks CENP-A^{Cnp1}, so that all associated nucleosomes contain H3. In contrast, at centromeres, when this same *cc2* DNA enters G2, it is assembled in chromatin in which approximately half the nucleosomes are placeholder H3 and the other half are CENP-A. The CENP-A N-terminal tail is distinct from that of H3 and lacks key lysine residues (K4, K36) whose modification in H3 aids transcription. Indeed, CENP-A nucleosomes inhibit transcription *in vitro* [75]. Therefore, the lower elongating RNAPII levels on centromeric *cc2* compared to ectopic *cc2* may be a consequence of CENP-A nucleosomes impeding transcription. The more easily detected elongating RNAPII on H3 chromatin-coated ectopic *cc2* centromere DNA during G2 may represent an extreme version of the G2 events that normally occur on centromere-located *cc2*. It seems likely that limited RNAPII transcription-coupled turnover also contributes to H3→CENP-A exchange at endogenous centromeres.

Several studies indicate that centromeric DNA is transcribed and linked with CENP-A deposition [44, 45]. It remains unclear whether the act of transcription, the resultant non-coding RNAs, or both are involved in promoting CENP-A assembly. Recent analysis indicates that human α satellite transcripts participate in CENP-A incorporation at centromeres and RNAPII-mediated transcription promotes CENP-A incorporation at *Drosophila* centromeres. Fission yeast central domain transcripts are exosome degraded, and consequently they are short lived and undetectable in wild-type cells [36]. However, we show that elongating RNAPII is clearly recruited to centromere DNA in G2 at the time of H3→CENP-A exchange. As at promoter-associated NDRs, fission yeast centromeric DNA has an intrinsic ability to recruit elongating RNAPII and destabilize H3 nucleosomes in a cell-cycle-regulated manner. Such embedded features may earmark these sequences for CENP-A^{Cnp1} incorporation.

Redundant processes are likely to mediate *de novo* assembly and maintenance of CENP-A on centromere DNA in order to ensure efficient and robust kinetochore formation. Different organisms may place more emphasis on different component processes involved in ensuring CENP-A chromatin assembly, but it seems likely that the inherent properties we have uncovered within fission yeast centromere DNA are also shared with transcribed centromeric DNA of other organisms. Despite the challenge of precisely assessing RNAPII engagement and histone dynamics at centromeres composed of highly repetitive DNA, it is now important to determine whether centromere DNA from other organisms also has an innate capacity to drive H3 eviction.

STAR★METHODS

Detailed methods are provided in the online version of this paper and include the following:

- KEY RESOURCES TABLE
- CONTACT FOR REAGENT AND RESOURCE SHARING
- EXPERIMENTAL MODEL AND SUBJECT DETAILS
  - Fission Yeast Methods
- METHOD DETAILS
  - Cytology
  - ChIP-qPCR
  - ChIP-Seq and ChIP-Nexus
  - Immunoprecipitation and Western analyses
  - RNA preparation and Reverse Transcriptase qPCR analysis
- QUANTIFICATION AND STATISTICAL ANALYSIS
- DATA AND SOFTWARE AVAILABILITY

SUPPLEMENTAL INFORMATION

Supplemental Information includes five figures and two tables and can be found with this article online at https://doi.org/10.1016/j.cub.2018.10.049.

ACKNOWLEDGMENTS

We are grateful to Fred van Leeuwen for RITE system constructs and the LoxP peptide antibody. We thank Nick Toda, Pauline Audergon, Jesus Torres-Garcia, Ryan Ard, Daniel Robertson, and David Kelly for their useful suggestions and technical input. M.S. was partly supported by an EMBO Long-Term Fellowship (ALTF 664-2011). P.T. was partly supported by funding from the European Commission Network of Excellence EpiGeneSys (HEALTH-F4-2010-257082) and a Wellcome Enhancement grant (095021) to R.C.A. S.C. was supported by a Wellcome PhD Studentship (086574). R.C.A. is a Wellcome Principal Research Fellow (200885); the Wellcome Centre for Cell Biology is supported by core funding from Wellcome (203149).

AUTHOR CONTRIBUTIONS

M.S. and R.C.A. jointly conceived the study. M.S., P.T., S.A.W., P.P.S., A.M.R., and S.C. performed experiments. M.S., P.T., and A.L.P. analyzed data. M.S. and R.C.A. wrote the manuscript with input from other authors.

CellPress

DECLARATION OF INTERESTS

The authors declare no competing interests.

Received: November 7, 2017
Revised: September 12, 2018
Accepted: October 22, 2018
Published: November 29, 2018

REFERENCES

1. Choo, K.H. (2001). Domain organization at the centromere and neocentromere. Dev. Cell *1*, 165–177.
2. Saunders, M., Fitzgerald-Hayes, M., and Bloom, K. (1988). Chromatin structure of altered yeast centromeres. Proc. Natl. Acad. Sci. USA *85*, 175–179.
3. Allshire, R.C., and Karpen, G.H. (2008). Epigenetic regulation of centromeric chromatin: old dogs, new tricks? Nat. Rev. Genet. *9*, 923–937.
4. Mendiburo, M.J., Padeken, J., Fülöp, S., Schepers, A., and Heun, P. (2011). *Drosophila* CENH3 is sufficient for centromere formation. Science *334*, 686–690.
5. Barnhart, M.C., Kuich, P.H.J.L., Stellfox, M.E., Ward, J.A., Bassett, E.A., Black, B.E., and Foltz, D.R. (2011). HJURP is a CENP-A chromatin assembly factor sufficient to form a functional de novo kinetochore. J. Cell Biol. *194*, 229–243.
6. Hori, T., Shang, W.-H., Takeuchi, K., and Fukagawa, T. (2013). The CCAN recruits CENP-A to the centromere and forms the structural core for kinetochore assembly. J. Cell Biol. *200*, 45–60.
7. Ishii, K., Ogiyama, Y., Chikashige, Y., Soejima, S., Masuda, F., Kakuma, T., Hiraoka, Y., and Takahashi, K. (2008). Heterochromatin integrity affects chromosome reorganization after centromere dysfunction. Science *321*, 1088–1091.
8. Shang, W.-H., Hori, T., Martins, N.M.C., Toyoda, A., Misu, S., Monma, N., Hiratani, I., Maeshima, K., Ikeo, K., Fujiyama, A., et al. (2013). Chromosome engineering allows the efficient isolation of vertebrate neocentromeres. Dev. Cell *24*, 635–648.
9. Scott, K.C., and Sullivan, B.A. (2014). Neocentromeres: a place for everything and everything in its place. Trends Genet. *30*, 66–74.
10. Sato, H., Masuda, F., Takayama, Y., Takahashi, K., and Saitoh, S. (2012). Epigenetic inactivation and subsequent heterochromatinization of a centromere stabilize dicentric chromosomes. Curr. Biol. *22*, 658–667.
11. Sullivan, B.A., and Schwartz, S. (1995). Identification of centromeric antigens in dicentric Robertsonian translocations: CENP-C and CENP-E are necessary components of functional centromeres. Hum. Mol. Genet. *4*, 2189–2197.
12. Kalitsis, P., and Choo, K.H.A. (2012). The evolutionary life cycle of the resilient centromere. Chromosoma *121*, 327–340.
13. Buscaino, A., Allshire, R., and Pidoux, A. (2010). Building centromeres: home sweet home or a nomadic existence? Curr. Opin. Genet. Dev. *20*, 118–126.
14. Lacoste, N., Woolfe, A., Tachiwana, H., Garea, A.V., Barth, T., Cantaloube, S., Kurumizaka, H., Imhof, A., and Almouzni, G. (2014). Mislocalization of the centromeric histone variant CenH3/CENP-A in human cells depends on the chaperone DAXX. Mol. Cell *53*, 631–644.
15. Heun, P., Erhardt, S., Blower, M.D., Weiss, S., Skora, A.D., and Karpen, G.H. (2006). Mislocalization of the *Drosophila* centromere-specific histone CID promotes formation of functional ectopic kinetochores. Dev. Cell *10*, 303–315.
16. Marshall, O.J., Chueh, A.C., Wong, L.H., and Choo, K.H.A. (2008). Neocentromeres: new insights into centromere structure, disease development, and karyotype evolution. Am. J. Hum. Genet. *82*, 261–282.
17. Ikeno, M., Grimes, B., Okazaki, T., Nakano, M., Saitoh, K., Hoshino, H., McGill, N.I., Cooke, H., and Masumoto, H. (1998). Construction of YAC-based mammalian artificial chromosomes. Nat. Biotechnol. *16*, 431–439.
18. Henikoff, S., Ahmad, K., and Malik, H.S. (2001). The centromere paradox: stable inheritance with rapidly evolving DNA. Science *293*, 1098–1102.
19. Baum, M., Ngan, V.K., and Clarke, L. (1994). The centromeric K-type repeat and the central core are together sufficient to establish a functional *Schizosaccharomyces pombe* centromere. Mol. Biol. Cell *5*, 747–761.
20. Okada, T., Ohzeki, J., Nakano, M., Yoda, K., Brinkley, W.R., Larionov, V., and Masumoto, H. (2007). CENP-B controls centromere formation depending on the chromatin context. Cell *131*, 1287–1300.
21. Alabert, C., and Groth, A. (2012). Chromatin replication and epigenome maintenance. Nat. Rev. Mol. Cell Biol. *13*, 153–167.
22. Shelby, R.D., Monier, K., and Sullivan, K.F. (2000). Chromatin assembly at kinetochores is uncoupled from DNA replication. J. Cell Biol. *151*, 1113–1118.
23. Mellone, B.G., Grive, K.J., Shteyn, V., Bowers, S.R., Oderberg, I., and Karpen, G.H. (2011). Assembly of *Drosophila* centromeric chromatin proteins during mitosis. PLoS Genet. *7*, e1002068.
24. Jansen, L.E., Black, B.E., Foltz, D.R., and Cleveland, D.W. (2007). Propagation of centromeric chromatin requires exit from mitosis. J. Cell Biol. *176*, 795–805.
25. Dunleavy, E.M., Beier, N.L., Gorgescu, W., Tang, J., Costes, S.V., and Karpen, G.H. (2012). The cell cycle timing of centromeric chromatin assembly in *Drosophila* meiosis is distinct from mitosis yet requires CAL1 and CENP-C. PLoS Biol. *10*, e1001460.
26. Schuh, M., Lehner, C.F., and Heidmann, S. (2007). Incorporation of *Drosophila* CID/CENP-A and CENP-C into centromeres during early embryonic anaphase. Curr. Biol. *17*, 237–243.
27. Lermontova, I., Schubert, V., Fuchs, J., Klatte, S., Macas, J., and Schubert, I. (2006). Loading of *Arabidopsis* centromeric histone CENH3 occurs mainly during G2 and requires the presence of the histone fold domain. Plant Cell *18*, 2443–2451.
28. Sullivan, K.F. (2001). A solid foundation: functional specialization of centromeric chromatin. Curr. Opin. Genet. Dev. *11*, 182–188.
29. Probst, A.V., Dunleavy, E., and Almouzni, G. (2009). Epigenetic inheritance during the cell cycle. Nat. Rev. Mol. Cell Biol. *10*, 192–206.
30. Dunleavy, E.M., Almouzni, G., and Karpen, G.H. (2011). H3.3 is deposited at centromeres in S phase as a placeholder for newly assembled CENP-A in G_1 phase. Nucleus *2*, 146–157.
31. Dunleavy, E.M., Roche, D., Tagami, H., Lacoste, N., Ray-Gallet, D., Nakamura, Y., Daigo, Y., Nakatani, Y., and Almouzni-Pettinotti, G. (2009). HJURP is a cell-cycle-dependent maintenance and deposition factor of CENP-A at centromeres. Cell *137*, 485–497.
32. Foltz, D.R., Jansen, L.E.T., Bailey, A.O., Yates, J.R., III, Bassett, E.A., Wood, S., Black, B.E., and Cleveland, D.W. (2009). Centromere-specific assembly of CENP-a nucleosomes is mediated by HJURP. Cell *137*, 472–484.
33. Hayashi, T., Fujita, Y., Iwasaki, O., Adachi, Y., Takahashi, K., and Yanagida, M. (2004). Mis16 and Mis18 are required for CENP-A loading and histone deacetylation at centromeres. Cell *118*, 715–729.
34. Schwartz, B.E., and Ahmad, K. (2005). Transcriptional activation triggers deposition and removal of the histone variant H3.3. Genes Dev. *19*, 804–814.
35. Chan, F.L., Marshall, O.J., Saffery, R., Kim, B.W., Earle, E., Choo, K.H., and Wong, L.H. (2012). Active transcription and essential role of RNA polymerase II at the centromere during mitosis. Proc. Natl. Acad. Sci. USA *109*, 1979–1984.
36. Choi, E.S., Strålfors, A., Castillo, A.G., Durand-Dubief, M., Ekwall, K., and Allshire, R.C. (2011). Identification of noncoding transcripts from within CENP-A chromatin at fission yeast centromeres. J. Biol. Chem. *286*, 23600–23607.
37. Choi, E.S., Strålfors, A., Catania, S., Castillo, A.G., Svensson, J.P., Pidoux, A.L., Ekwall, K., and Allshire, R.C. (2012). Factors that promote H3 chromatin integrity during transcription prevent promiscuous deposition of CENP-A(Cnp1) in fission yeast. PLoS Genet. *8*, e1002985.

38. Catania, S., Pidoux, A.L., and Allshire, R.C. (2015). Sequence features and transcriptional stalling within centromere DNA promote establishment of CENP-A chromatin. PLoS Genet. *11*, e1004986.
39. Chen, C.-C., Bowers, S., Lipinszki, Z., Palladino, J., Trusiak, S., Bettini, E., Rosin, L., Przewloka, M.R., Glover, D.M., O'Neill, R.J., and Mellone, B.G. (2015). Establishment of centromeric chromatin by the CENP-A assembly factor CAL1 requires FACT-mediated transcription. Dev. Cell *34*, 73–84.
40. Rošić, S., Köhler, F., and Erhardt, S. (2014). Repetitive centromeric satellite RNA is essential for kinetochore formation and cell division. J. Cell Biol. *207*, 335–349.
41. Grenfell, A.W., Heald, R., and Strzelecka, M. (2016). Mitotic noncoding RNA processing promotes kinetochore and spindle assembly in *Xenopus*. J. Cell Biol. *214*, 133–141.
42. Molina, O., Vargiu, G., Abad, M.A., Zhiteneva, A., Jeyaprakash, A.A., Masumoto, H., Kouprina, N., Larionov, V., and Earnshaw, W.C. (2016). Epigenetic engineering reveals a balance between histone modifications and transcription in kinetochore maintenance. Nat. Commun. *7*, 13334.
43. Topp, C.N., Zhong, C.X., and Dawe, R.K. (2004). Centromere-encoded RNAs are integral components of the maize kinetochore. Proc. Natl. Acad. Sci. USA *101*, 15986–15991.
44. McNulty, S.M., Sullivan, L.L., and Sullivan, B.A. (2017). Human centromeres produce chromosome-specific and array-specific alpha satellite transcripts that are complexed with CENP-A and CENP-C. Dev. Cell *42*, 226–240.e6.
45. Bobkov, G.O.M., Gilbert, N., and Heun, P. (2018). Centromere transcription allows CENP-A to transit from chromatin association to stable incorporation. J. Cell Biol. *217*, 1957–1972.
46. Carroll, C.W., Silva, M.C.C., Godek, K.M., Jansen, L.E.T., and Straight, A.F. (2009). Centromere assembly requires the direct recognition of CENP-A nucleosomes by CENP-N. Nat. Cell Biol. *11*, 896–902.
47. Carroll, C.W., Milks, K.J., and Straight, A.F. (2010). Dual recognition of CENP-A nucleosomes is required for centromere assembly. J. Cell Biol. *189*, 1143–1155.
48. Smoak, E.M., Stein, P., Schultz, R.M., Lampson, M.A., and Black, B.E. (2016). Long-term retention of CENP-A nucleosomes in mammalian oocytes underpins transgenerational inheritance of centromere identity. Curr. Biol. *26*, 1110–1116.
49. Partridge, J.F., Borgstrøm, B., and Allshire, R.C. (2000). Distinct protein interaction domains and protein spreading in a complex centromere. Genes Dev. *14*, 783–791.
50. Allshire, R.C., and Ekwall, K. (2015). Epigenetic regulation of chromatin states in *Schizosaccharomyces pombe*. Cold Spring Harb. Perspect. Biol. *7*, a018770.
51. Takahashi, K., Murakami, S., Chikashige, Y., Funabiki, H., Niwa, O., and Yanagida, M. (1992). A low copy number central sequence with strict symmetry and unusual chromatin structure in fission yeast centromere. Mol. Biol. Cell *3*, 819–835.
52. Takayama, Y., Sato, H., Saitoh, S., Ogiyama, Y., Masuda, F., and Takahashi, K. (2008). Biphasic incorporation of centromeric histone CENP-A in fission yeast. Mol. Biol. Cell *19*, 682–690.
53. Lando, D., Endesfelder, U., Berger, H., Subramanian, L., Dunne, P.D., McColl, J., Klenerman, D., Carr, A.M., Sauer, M., Allshire, R.C., et al. (2012). Quantitative single-molecule microscopy reveals that CENP-A(Cnp1) deposition occurs during G2 in fission yeast. Open Biol. *2*, 120078.
54. Takahashi, K., Chen, E.S., and Yanagida, M. (2000). Requirement of Mis6 centromere connector for localizing a CENP-A-like protein in fission yeast. Science *288*, 2215–2219.
55. Verzijlbergen, K.F., Menendez-Benito, V., van Welsem, T., van Deventer, S.J., Lindstrom, D.L., Ovaa, H., Neefjes, J., Gottschling, D.E., and van Leeuwen, F. (2010). Recombination-induced tag exchange to track old and new proteins. Proc. Natl. Acad. Sci. USA *107*, 64–68.
56. Hagan, I.M., Grallert, A., and Simanis, V. (2016). Synchronizing progression of *Schizosaccharomyces pombe* cells from G2 through repeated rounds of mitosis and S phase with cdc25-22 arrest release. Cold Spring Harb. Protoc. *2016*, pdb.prot091264.
57. Ahmad, K., and Henikoff, S. (2002). Histone H3 variants specify modes of chromatin assembly. Proc. Natl. Acad. Sci. USA *99* (*Suppl 4*), 16477–16484.
58. Pidoux, A.L., Choi, E.S., Abbott, J.K.R., Liu, X., Kagansky, A., Castillo, A.G., Hamilton, G.L., Richardson, W., Rappsilber, J., He, X., and Allshire, R.C. (2009). Fission yeast Scm3: a CENP-A receptor required for integrity of subkinetochore chromatin. Mol. Cell *33*, 299–311.
59. Williams, J.S., Hayashi, T., Yanagida, M., and Russell, P. (2009). Fission yeast Scm3 mediates stable assembly of Cnp1/CENP-A into centromeric chromatin. Mol. Cell *33*, 287–298.
60. He, Q., Johnston, J., and Zeitlinger, J. (2015). ChIP-nexus enables improved detection of *in vivo* transcription factor binding footprints. Nat. Biotechnol. *33*, 395–401.
61. Aygün, O., Mehta, S., and Grewal, S.I. (2013). HDAC-mediated suppression of histone turnover promotes epigenetic stability of heterochromatin. Nat. Struct. Mol. Biol. *20*, 547–554.
62. Svensson, J.P., Shukla, M., Menendez-Benito, V., Norman-Axelsson, U., Audergon, P., Sinha, I., Tanny, J.C., Allshire, R.C., and Ekwall, K. (2015). A nucleosome turnover map reveals that the stability of histone H4 Lys20 methylation depends on histone recycling in transcribed chromatin. Genome Res. *25*, 872–883.
63. Blower, M.D. (2016). Centromeric transcription regulates Aurora-B localization and activation. Cell Rep. *15*, 1624–1633.
64. Castillo, A.G., Mellone, B.G., Partridge, J.F., Richardson, W., Hamilton, G.L., Allshire, R.C., and Pidoux, A.L. (2007). Plasticity of fission yeast CENP-A chromatin driven by relative levels of histone H3 and H4. PLoS Genet. *3*, e121.
65. Pidoux, A.L., Richardson, W., and Allshire, R.C. (2003). Sim4: a novel fission yeast kinetochore protein required for centromeric silencing and chromosome segregation. J. Cell Biol. *161*, 295–307.
66. Santos, A., Wernersson, R., and Jensen, L.J. (2015). Cyclebase 3.0: a multi-organism database on cell-cycle regulation and phenotypes. Nucleic Acids Res. *43*, D1140–D1144.
67. Fujita, Y., Hayashi, T., Kiyomitsu, T., Toyoda, Y., Kokubu, A., Obuse, C., and Yanagida, M. (2007). Priming of centromere for CENP-A recruitment by human hMis18alpha, hMis18beta, and M18BP1. Dev. Cell *12*, 17–30.
68. Maddox, P.S., Hyndman, F., Monen, J., Oegema, K., and Desai, A. (2007). Functional genomics identifies a Myb domain-containing protein family required for assembly of CENP-A chromatin. J. Cell Biol. *176*, 757–763.
69. Castillo, A.G., Pidoux, A.L., Catania, S., Durand-Dubief, M., Choi, E.S., Hamilton, G., Ekwall, K., and Allshire, R.C. (2013). Telomeric repeats facilitate CENP-A(Cnp1) incorporation via telomere binding proteins. PLoS ONE *8*, e69673.
70. Bodor, D.L., Mata, J.F., Sergeev, M., David, A.F., Salimian, K.J., Panchenko, T., Cleveland, D.W., Black, B.E., Shah, J.V., and Jansen, L.E. (2014). The quantitative architecture of centromeric chromatin. eLife *3*, e02137.
71. Harrington, J.J., Van Bokkelen, G., Mays, R.W., Gustashaw, K., and Willard, H.F. (1997). Formation of *de novo* centromeres and construction of first-generation human artificial microchromosomes. Nat. Genet. *15*, 345–355.
72. Venkatesh, S., and Workman, J.L. (2015). Histone exchange, chromatin structure and the regulation of transcription. Nat. Rev. Mol. Cell Biol. *16*, 178–189.
73. Schwabish, M.A., and Struhl, K. (2004). Evidence for eviction and rapid deposition of histones upon transcriptional elongation by RNA polymerase II. Mol. Cell. Biol. *24*, 10111–10117.
74. Kulaeva, O.I., Hsieh, F.-K., and Studitsky, V.M. (2010). RNA polymerase complexes cooperate to relieve the nucleosomal barrier and evict histones. Proc. Natl. Acad. Sci. USA *107*, 11325–11330.
75. Shandilya, J., Senapati, P., Hans, F., Menoni, H., Bouvet, P., Dimitrov, S., Angelov, D., and Kundu, T.K. (2014). Centromeric histone variant CENP-A

repesses acetylation-dependent chromatin transcription that is relieved by histone chaperone NPM1. J. Biochem. *156*, 221–227.

76. Langmead, B., and Salzberg, S.L. (2012). Fast gapped-read alignment with Bowtie 2. Nat. Methods *9*, 357–359.

77. Zhang, Y., Liu, T., Meyer, C.A., Eeckhoute, J., Johnson, D.S., Bernstein, B.E., Nusbaum, C., Myers, R.M., Brown, M., Li, W., and Liu, X.S. (2008). Model-based analysis of ChIP-seq (MACS). Genome Biol. *9*, R137.

78. Ramírez, F., Dündar, F., Diehl, S., Grüning, B.A., and Manke, T. (2014). deepTools: a flexible platform for exploring deep-sequencing data. Nucleic Acids Res. *42*, W187–W191.

79. Wang, L., Chen, J., Wang, C., Uusküla-Reimand, L., Chen, K., Medina-Rivera, A., Young, E.J., Zimmermann, M.T., Yan, H., Sun, Z., et al. (2014). MACE: model based analysis of ChIP-exo. Nucleic Acids Res. *42*, e156.

80. Moreno, S., Klar, A., and Nurse, P. (1991). Molecular genetic analysis of fission yeast *Schizosaccharomyces pombe*. Methods Enzymol. *194*, 795–823.

81. Pidoux, A.L., Uzawa, S., Perry, P.E., Cande, W.Z., and Allshire, R.C. (2000). Live analysis of lagging chromosomes during anaphase and their effect on spindle elongation rate in fission yeast. J. Cell Sci. *113*, 4177–4191.

STAR★METHODS

KEY RESOURCES TABLE

REAGENT or RESOURCE	SOURCE	IDENTIFIER
Antibodies		
Rabbit polyclonal anti-HA	Abcam	Cat#ab9110; RRID:AB_307019
Mouse monoclonal anti-HA 12CA5	In-house preparation	N/A
Mouse monoclonal anti-T7	Merck	Cat#69522; RRID:AB_11211744
Donkey anti-Rabbit Alexa 594	ThermoFisher Scientific	Cat#A21207; RRID:AB_141637
Donkey anti-Mouse Alexa 488	ThermoFisher Scientific	Cat#A21202; RRID:AB_141607
Sheep polyclonal anti-Cnp1	In-house preparation	N/A
Rabbit polyclonal anti-H3	Abcam	Cat#ab1791; RRID:AB_302613
Rabbit monoclonal anti-H4	Merck	Cat#05-858; RRID:AB_390138
Goat polyclonal anti-T7	Abcam	Cat#ab9138; RRID:AB_307038
Rabbit polyclonal anti-GFP	ThermoFisher Scientific	Cat#A11122; RRID:AB_221569
Rabbit polyclonal anti-Phospho serine 2 RNA polymerase II	Abcam	Cat#ab5095; RRID:AB_304749
Mouse monoclonal anti-GFP antibody	Sigma-Aldrich /Roche	Cat#11814460001; RRID:AB_390913
IRDye 680RD anti-Rabbit	Li-Cor	Cat#926-68073; RRID:AB_10954442
IRDye 800CW anti-Mouse	Li-Cor	Cat#926-32210; RRID:AB_621842
Rat monoclonal anti-HA clone 3F10	Sigma-Aldrich /Roche	Cat#11867423001; RRID:AB_390918
Rabbit polyclonal anti-LoxP	Gift from Fred van Leeuwen [55],	N/A
Anti-Rat IgG HRP	Sigma-Aldrich	Cat#A9037; RRID:AB_258429
Anti-Rabbit IgG HRP	Sigma-Aldrich	Cat#A6154; RRID:AB_258284
Bacterial and Virus Strains		
NEB 5-alpha Competent *E. coli* (High Efficiency)	New England Biolabs	Cat#C2987H
Chemicals, Peptides, and Recombinant Proteins		
β-Estradiol	Sigma-Aldrich	Cat#E2758
DMP (dimethyl pimelimidate)	ThermoFisher Scientific	Cat#21666
Critical Commercial Assays		
QIAquick PCR Purification Kit	QIAGEN	Cat#28104
Light Cycler 480 SybrGreen Master Mix	Roche	Cat#04887352001
NEXTflex-96-DNA barcodes	Bioo Scientific	Cat#514105
RNeasy Mini kit	QIAGEN	Cat#74104
SuperScript IV First-Strand Synthesis System	ThermoFisher Scientific	Cat#18091050
Deposited Data		
Sequencing data and analyses	This paper	GEO: GSE106494
Experimental Models: Organisms/Strains		
Fission Yeast strains	NA	See Table S1
Oligonucleotides		
Oligonucleotides	This paper	See Table S2
Recombinant DNA		
pTW040	Gift from Fred van Leeuwen [55],	N/A
pFvL118/119	Gift from Fred van Leeuwen [55],	N/A
pTW081	Gift from Fred van Leeuwen [55],	N/A
pRAD11-CreEBD	This paper	N/A
pRAD13-CreEBD	This paper	N/A

(*Continued on next page*)

Continued		
REAGENT or RESOURCE	SOURCE	IDENTIFIER
Software and Algorithms		
Bowtie2	[76]	http://bowtie-bio.sourceforge.net/bowtie2/index.shtml
Macs2	[77]	https://pypi.org/project/MACS2/
Deeptools	[78]	https://pypi.org/project/deepTools/
MACE	[79]	https://sourceforge.net/projects/chipexo/files/MACE-1.2.tar.gz/download
Other		
Protein G-dynabeads	ThermoFisher Scientific	Cat#10009D
Protein G-Agarose	Sigma-Aldrich/Roche	Cat#11243233001
Halt protease and phosphatase inhibitor	ThermoFisher Scientific	Cat#1861281
Micrococcal nuclease	Sigma-Aldrich	Cat#N3755
Quick blunting kit	New England Biolabs	Cat#E1201L
Klenow exo$^-$	New England Biolabs	Cat#M0212S
T4 DNA polymerase	New England Biolabs	Cat#M0203S
DNA polymerase I large fragment	New England Biolabs	Cat#M0210S
T4 polynucleotide kinase	New England Biolabs	Cat#M0201S
Lambda exonuclease	New England Biolabs	Cat#M0262S
RecJf	New England Biolabs	Cat# M0264L
Circligase	Epicenter	Cat#CL4111K
Agencourt Ampure XP beads	Beckman Coulter Life Sciences	Cat#A63881

CONTACT FOR REAGENT AND RESOURCE SHARING

Further information and requests for resources and reagents should be directed to and will be fulfilled by the Lead Contact, Robin Allshire (Robin.Allshire@ed.ac.uk).

EXPERIMENTAL MODEL AND SUBJECT DETAILS

Fission Yeast Methods

Standard genetic and molecular techniques were followed. Fission yeast methods were as described [80]. YES (Yeast Extract with Supplements) was used as a rich medium or PMG (Pombe Minimal Glutamate) for growth of cells in liquid cultures. 4X YES was used for experiments where higher cell numbers were required. CENP-A^{Cnp1} and H3.2 RITE strains were constructed by PCR amplifying HA/T7 or T7/HA RITE cassettes described in [55] and integration at the endogenous gene locus. Cre-EBD open reading frame was PCR amplified from pTW040 [55] and cloned in pRAD11, 13, 15 vectors containing different strengths of ADH promoters (Gift from Y. Watanabe). The Cre-EBD plasmids were integrated at the *ars1* locus by transformation of the plasmid DNAs linearized by *Mlu*I digestion. Strains are described in Table S1.

METHOD DETAILS

Cytology

Cells were fixed with 3.7% formaldehyde for 7 min at room temperature. Immuno-localization staining was performed as described [81]. The following antibodies were used at 1:100 dilution: Anti HA (Abcam, ab9110), anti-T7 (Merck, 69522); Alexa 594 and 488 labeled secondary antibodies at 1:1000 dilution (Life Technologies). Single images were acquired with a Zeiss LSM 880 confocal microscope equipped with Airyscan superresolution imaging module, using a 100X/1.40 NA Plan-Apochromat Oil DIC M27 objective lens. Images were processed using ZEN Black image acquisition and processing software (Zeiss MicroImaging). Images were analyzed using ImageJ as follows; the region of interest was selected by the user (CENP-A^{Cnp1} nuclear spot) in both HA and T7 channels by manually intensity thresholding the image. The pixel intensities from the thresholded area were then calculated to give mean intensities. For each time point, (except T5 where n = 94), 100 cells were analyzed and intensity for each signal either HA (old CENP-A) or T7 (new CENP-A) is presented as a boxplot.

ChIP-qPCR

Cells were fixed with 1% formaldehyde for 15 min followed by quenching with 250 mM Glycine for 5 min at room temperature. ChIP was essentially performed as described [64] using antibodies against CENP-A^{Cnp1} (Sheep in-house; 10 μl), H3 (ab1791, Abcam; 2 μl), H4 (Merck, 05-858; 2 μl), HA (12CA5, in-house preparation; 2 μl), T7 (Abcam, ab9138; 2 μl), GFP (ThermoFisher Scientific, A11122; 2 μl) and Phopho serine 2 RNA polymerase II (Abcam, ab5095; 2 μl). 2.5×10^8 cells were used per ChIP sample. Where indicated, formaldehyde fixed *SchizoSaccharomyces octosporus* cells were added at the cell lysis stage for spiked-in control. Quantitative PCR reactions were performed in 10 μL volume with Light Cycler 480 SybrGreen Master Mix (Roche, 04887352001). The data were analyzed using Light Cycler 480 Software 1.5 (Roche). q-PCR primers are listed in Table S2. Percentage immunoprecipitation values were calculated using the equation: $\{2\text{^}-(Cp^{IP}-Cp^{Input})\}*100$. Cp^{Input} values were adjusted for the amount of lysate and dilution used in qPCR. Where mentioned, normalized percentage IP values were obtained by dividing all the % IP values in the respective time point series with the % IP value obtained from the indicated time point (specified in the figure legend).

ChIP-Seq and ChIP-Nexus

Due to higher number of cells required, for ChIP-Seq from synchronized cell cultures, cells were grown in 4X-YES and ChIP protocol was modified. Briefly, cell pellets corresponding to 7.5×10^8 cells were lysed by four 1-minute cycles of bead beating in 500 μL of lysis buffer (50 mM HEPES-KOH, pH 7.5, 140 mM NaCl, 1cmM EDTA, 1% Triton X-100, 0.1% sodium deoxycholate). Insoluble chromatin fraction was isolated by centrifugation at 6000 *g*. The pellet was washed with 1 mL lysis buffer. This washed pellet was gently resuspended in 300 μL lysis buffer containing 0.2% SDS and sheared by sonication with Bioruptor (Diagenode) for 30 min (30 s On, 30 s off at high setting). 900 μL of lysis buffer (without SDS) was added and samples were clarified by centrifugation at 17000 *g* for 20 min. Supernatants were used for ChIP. Respective antibody and protein G-dynabeads (ThermoFisher Scientific) amounts were scaled up according to the cell number. Immunoprecipitated DNA was recovered using QIAGEN PCR purification kit. ChIP-Seq libraries were prepared with 1-5 ng of ChIP or 10 ng of input DNA. DNA was end-repaired using NEB Quick blunting kit (E1201L). The blunt, phosphorylated ends were treated with Klenow exo^- (NEB, M0212S) and dATP to yield a protruding 3- ‘A’ base for ligation of NEXTflex adapters (Bioo Scientific) which have a single ‘T’ base overhang at the 3′ end. After adaptor ligation DNA was PCR amplified with Illumina primers for 13-15 cycles and library fragments of ~300 bp (insert plus adaptor sequences) were selected using Ampure XP beads.

ChIP-Nexus libraries were prepared essentially as described [60]. Briefly, protein G-dynabeads bound DNA-protein-complexes were affinity selected using antibodies. DNA was end repaired using T4 DNA polymerase (NEB, M0203S), DNA polymerase I large fragment (NEB, M0210S) and T4 polynucleotide kinase (NEB, M0201S). A single 3′-A overhang was added using Klenow exo^- polymerase. Adapters were ligated and blunted again by Klenow exo^- polymerase to fill in the 5′ overhang first and then by T4 DNA polymerase to trim possible 3′ overhangs. Blunted DNA was then sequentially digested by lambda exonuclease (NEB, M0262S) and RecJf (NEB, M0264L). Digested single strand DNA was then eluted, reverse cross-linked and phenol-chloroform extracted. Fragments were then self-circularized by Circligase (Epicenter, CL4111K). An oligonucleotide was hybridized to circularized single DNA for subsequent BamHI digestion in order to linearize the DNA. This linearized single strand DNA was then PCR-amplified using adaptor sequences and libraries were purified and size selected using Ampure XP beads. The libraries were sequenced following Illumina HiSeq2500 work flow.

Next generation sequencing libraries were aligned to *S. pombe* build ASM294v2.20 using Bowtie2 [76]. ChIP-Seq reads with mapping qualities lower than 30, and read pairs mapped over 500-nt apart or less than 100-nt, were discarded. All the ChIP-Seq data were normalized with respect to their input data (Enrichment = $IP_{RPKM}/Input_{RPKM}$). ChIP peaks were identified from the alignments using Macs2 [77] with the corresponding input data. Deeptools [78] was used to generate genome wide enrichment profiles using a 50 bp window size and the data visualized using the IGV genome browser. ChIP-Nexus data were analyzed using MACE [79]. ChIP-Nexus data described in Figures 4F and S4 were normalized with their input data.

Immunoprecipitation and Western analyses

Cell were grown in 4X-YES and 5×10^9 cells were used per IP. Briefly, cells were harvested by centrifugation at 3500*g*, washed twice with water and flash frozen in liquid nitrogen. Frozen cell pellets were ground using Retsch MM400 mill. The grindate was resuspended in lysis buffer (10 mM Tris pH7.4, 5 mM $CaCl_2$, 5 mM $MgCl_2$, 50 mM NaCl, 0.1% IGEPAL-CA630 and supplemented with Halt protease and phosphatase inhibitor (ThermoFisher Scientific, 1861281) and 2 mM PMSF. Chromatin was solubilized by incubation with 2 units of Micrococcal nuclease (Sigma, N3755) for 10 min at 37°C. MNase digestion was stopped by adding EGTA to 20 mM and lysate were rotated at 4°C for 1 hr to ensure chromatin solubilization. Lysates were clarified by centrifugation at 20,000*g* for 10 min and supernatants were used for immunoprecipitation. Cleared lysates were incubated with 10 μg of anti-GFP antibody (Roche, 11814460001) and 25 μL of protein G-dynabeads (ThermoFisher Scientific), which were already crosslinked with DMP (dimethyl pimelimidate) (ThermoFisher Scientific, 21666), for 1 hr at 4°C with gentle rotation. Bead-bound affinity-selected chromatin was washed three times with lysis buffer and eluted with LDS loading buffer (ThermoFisher Scientific, 84788). Western blotting detection was performed using anti-GFP (Roche, 11814460001), anti-H3 (Abcam, ab1791) and anti-phospho-Serine2-RNA polymerase II (Abcam, ab5095) and secondary IRDye 680RD anti-Rabbit (Li-Cor, 926-68073) and IRDye 800CW anti-Mouse antibodies (Li-Cor, 926-32210).

CENP-A^{cnp1} RITE strain (T7 to HA) Cell population were synchronized using *cdc25-22* block release. Tag switch was induced during the block by addition of β-estradiol to 1 μM for the last 2 hr of the block. Cell populations were synchronously released from the G2

block by lowering the culture temperature to 25°C and culture samples were collected at indicated time intervals. $7.5x10^8$ cells were used for each immunoprecipitation sample. Cells were washed twice with water and frozen. Cell pellets were lysed by bead beating in 1.5 mL lysis buffer (50 mM HEPES-KOH, pH7.5, 140 mM NaCl, 1mM EDTA, 1% Triton X-100, 0.1% Sodium Deoxycholate) containing 1mM PMSF and protease inhibitors (Roche). Cell lysates were sonicated for 12 cycles (30 s on, 30 s off) using a Bioruptor (Diagenode), rotated for 1 hr at 4°C and clarified by centrifugation at 17000 g for 20 min. Cleared lysates were incubated overnight with 10 μL CENP-A^{Cnp1} anti-serum and 50 μL of protein-G agarose beads (Roche). Beads were washed 3 times with the lysis buffer and immunoprecipitated material was eluted by incubating the beads in 1XLDS loading buffer (ThermoFisher Scientific). Western analysis was performed using anti-HA (Sigma, 11867423001, clone 3F10), anti-LoxP (gift from Fred van Leeuwen), secondary anti-Rat IgG HRP (Sigma, A9037) and anti-Rabbit IgG HRP (Sigma, A6154).

RNA preparation and Reverse Transcriptase qPCR analysis

RNA was extracted from $2x10^7$ synchronized CENP-A^{cnp1} RITE strain (HA to T7) at indicated time points using RNeasy Mini kit (QIAGEN, 74104) according to manufacturer's instruction. 2 μg of RNA was converted to cDNAs using SuperScript IV First-Strand Synthesis System (ThermoFisher Scientific, 18091050). Quantitative PCR reactions were performed in 10 μL volume with Light Cycler 480 SybrGreen Master Mix (Roche, 04887352001). The data were analyzed using Light Cycler 480 Software 1.5 (Roche). q-PCR primers are listed in Table S2.

QUANTIFICATION AND STATISTICAL ANALYSIS

Statistical parameters are described in the relevant figure legends. Where indicated, the value of n represents the number of independent replicates. Histograms and line plots shown in the study represent mean values from indicated number of replicates. Error bars represent the calculated standard deviation.

In Figures 1D, 4F, 5D, and S4, standardized box-plots are shown. The box includes data in the interquartile range, IQR: 25th-75th. The middle lines represent the calculated medians. Whisker lengths represent the lowest and highest data range within 1.5 IQR from the box. Outlier values are shown as circles. In Figures 6C and S5B, violin plots are used to visualize the distribution of the data: black bars in the center represents the IQR, white circles represent the calculated median values. The thin black line shows the 95% confidence intervals. A mPearson's correlation test was performed on H3 and H4 ChIP-Nexus experimental replicates (r value > 0.98).

DATA AND SOFTWARE AVAILABILITY

The accession number for the sequencing data reported in this paper is GEO: GSE106494.

Current Biology, Volume *28*

Supplemental Information

Centromere DNA Destabilizes H3 Nucleosomes

to Promote CENP-A Deposition during the Cell Cycle

Manu Shukla, Pin Tong, Sharon A. White, Puneet P. Singh, Angus M. Reid, Sandra Catania, Alison L. Pidoux, and Robin C. Allshire

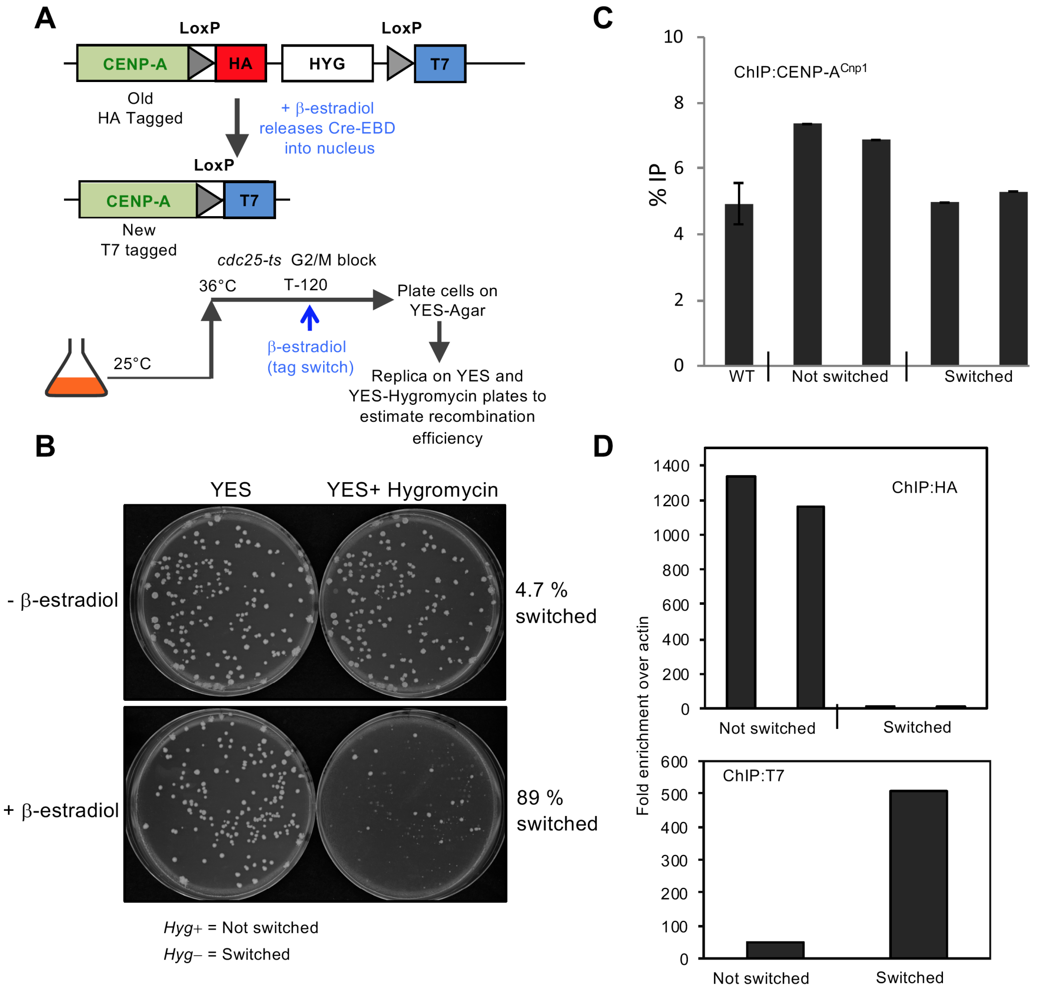

Figure S1. Validation of Recombination Induced Tag Exchange (RITE) system in *S. pombe*. Related to figure 1.

(A) Schematics of the CENP-A^{Cnp1} RITE experiment. *cdc25-22* cells were blocked in G2 and tag switch was induced by addition of β-estradiol as described in Figure 1A. After 2 hours of β-estradiol treatment, cells were plated on non-selective YES agar plates. Cre-EBD induced recombination results in tag switch accompanied by loss of *hygMX6* marker. Colonies were replica plated on YES agar plates with or without hygromycin to ascertain efficiency of tag switch.

(B) Representative experiment showing efficiency of tag switching. Percentage of *hyg*$^+$ colonies with or without β-estradiol treatment is shown.

(C) Centromeric CENP-A^{Cnp1} levels are not affected by presence of C-terminal RITE tags. CENP-A^{Cnp1} ChIP was performed on Non-switched (*hyg*$^+$) and switched (*hyg*$^-$) RITE tagged cell colonies (from B). Untagged wild type strain was used as control (Error bar represents mean ±SD, n=3). Y axis shows percentage immunoprecipitation values at central core 2 (*cc2*).

(D) Verification of tag switch by ChIP. Non-switched (*hyg*$^+$) and switched (*hyg*$^-$) RITE tagged cell colonies (from B) were grown in liquid culture and ChIPs for HA and T7 epitopes were performed. ChIP results show loss of HA tag (top panel) and presence of T7 tag after RITE.

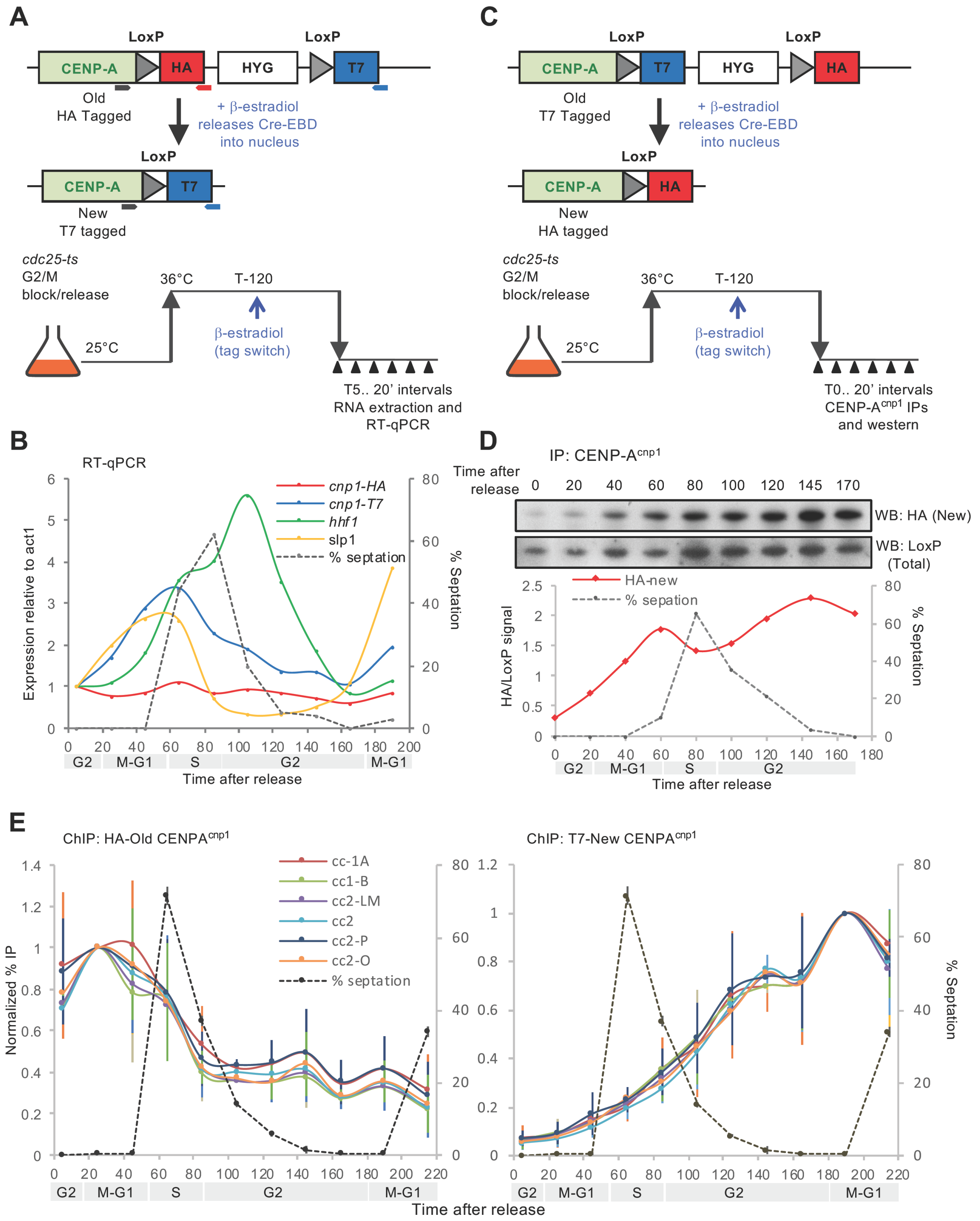

Figure S2. New CENP-A^{Cnp1} is expressed prior to its deposition at centromeres in G2. Related to Figure 1.

(A) Diagram of Recombination Induced Tag Exchange (RITE) system and CENP-A^{Cnp1}-RITE tag swap. *cdc25-22* ts mutant cells were blocked in G2 by incubation at 36°C, tag swap was induced by β-estradiol addition. Cells were released synchronously into the cell cycle by shifting to 25°C. Samples for RNA were collected at indicated time-points (T5-T190). Locations of common *cnp1*$^{+}$, HA and T7 tag specific primers are indicated.

(B) RT-qPCR for expression kinetics of *cnp1-HA* or tag swapped *cnp1-T7* genes through cell cycle. Cell cycle dependent exemplar genes *slp1+* (mitosis) and *hhf1*$^{+}$ (S-phase) are shown. Data were normalized to *act1+* expression levels for corresponding time points. Percentage of septated cells and corresponding cell cycle stages are indicated.

(C) Diagram of Recombination Induced Tag Exchange (RITE) system and CENP-A^{Cnp1}-RITE tag swap. *cdc25-22* ts mutant cells were blocked in G2 by incubation at 36°C, tag swap was induced by β-estradiol addition. Cells were released synchronously into the cell cycle by shifting to 25°C. Samples for CENP-A^{cnp1} immunoprecipitations were collected at indicated time-points (T0-T170). CENP-A^{Cnp1} affinity selection was performed using anti-CENP-A^{Cnp1} serum and immunoprecipitated material was probed with anti-LoxP peptide antibody (for detection of total CENP-A^{cnp1}) and anti-HA peptide antibody (for detection of new CENP-A^{cnp1}).

(D) Western blot showing levels of total CENP-A^{Cnp1} (LoxP) and new CENP-A^{Cnp1} (HA). Densitometry was performed using ImageJ and HA western signals were normalized to total CENP-A^{cnp1} (LoxP western) signal for corresponding time points. % septation and cell cycle phases are indicated.

(E) ChIP-qPCR analysis showing the profiles for HA tagged old (left panel) and T7 tagged new (right panel) CENP-A^{Cnp1} during cell cycle at various locations on central core 1 and 2 (related to figure 1B). %IP values were normalized with time point 'T25' values for HA ChIPs and time point 'T190' values for T7 ChIPs respectively. Data shown here are average values from two independent experiments. Error bars represent mean ± SD. Percentage of septated cells is shown to follow the progress of the cell cycle.

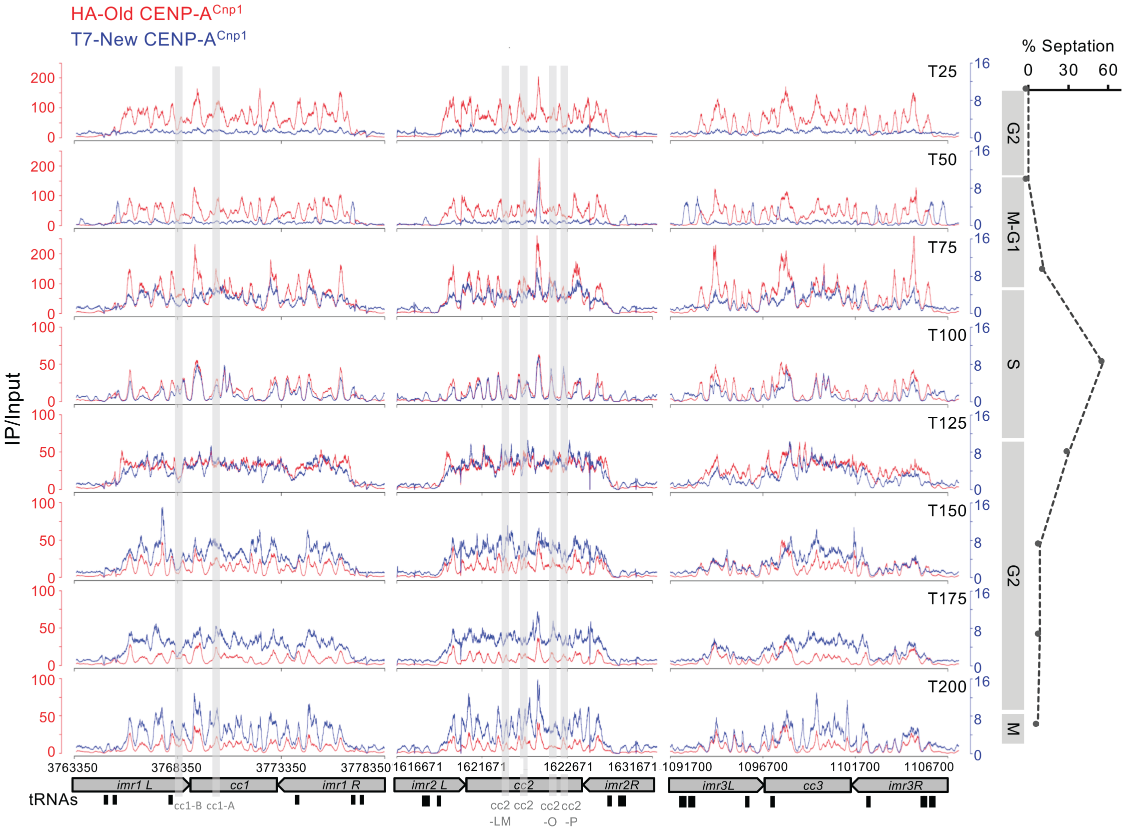

Figure S3. New and old CENP-A^{Cnp1} profiles at fission yeast centromeres through the cell-cycle. Related to figure 2.

ChIP-Seq profiles for HA tagged old (in red) and T7 tagged new (in blue) CENP-A^{Cnp1} through the cell cycle at central core 1 (left panel), central core 2 (middle panel) and central core 3 (right panel). Experimental scheme is same as described in Figure 1A. Respective fold enrichment values (IP relative to Input) for HA (red) and T7 (blue) are shown on Y axes. Corresponding time points, approximate cell cycle phase and chromosomal locations are indicated. Primer locations used in qChIP experiments (Figure 1B and S2E) are indicated by grey bars.

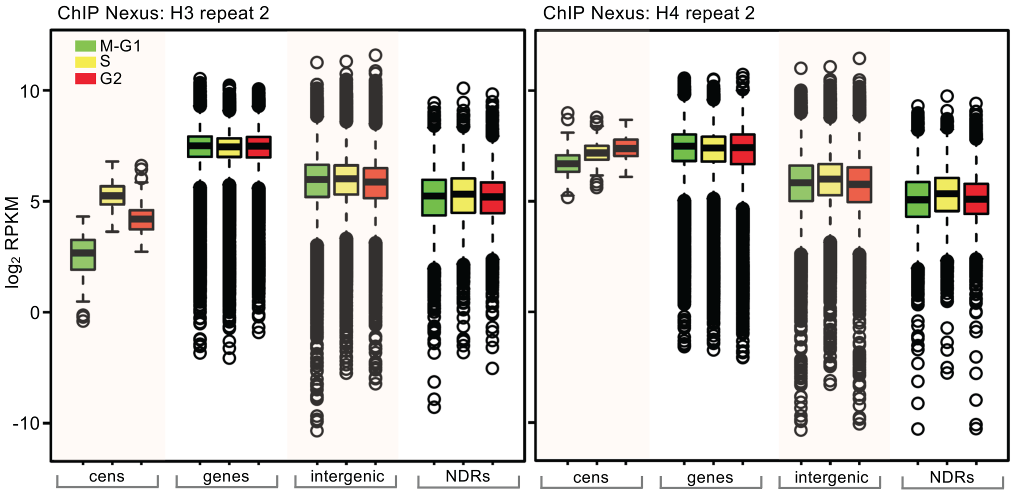

Figure S4. Histone H3 levels increase at centromeres during S-phase. Related to figure 4.

Quantitation of H3 and H4 occupancy by ChIP-Nexus in representative mitosis/G1, S and G2 cell cycle phase samples (replicate 2). Box plot of H3 (left panel) and H4 occupancy levels (right panel) over central core DNA (*cc1*, *cc2*, *cc3* together), gene bodies, intergenic regions and NDRs. Y axis shows log_2 RPKM values for corresponding genomic locations.

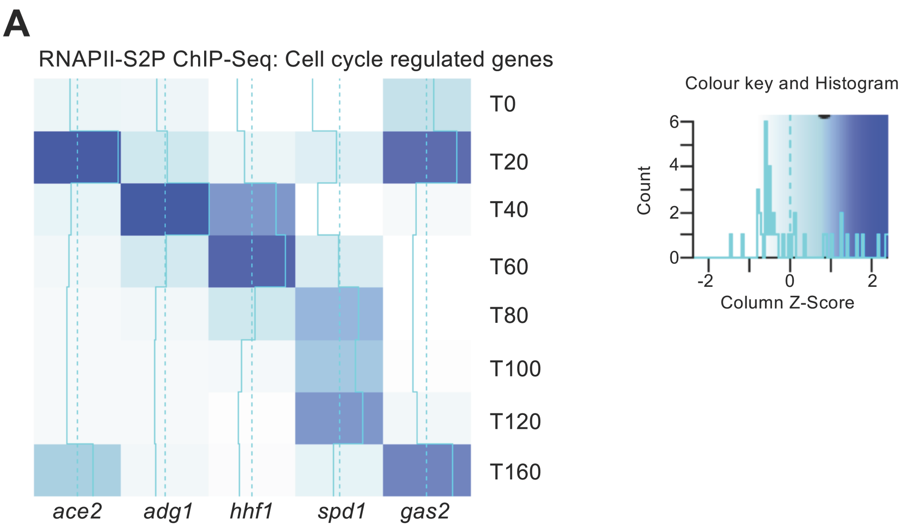

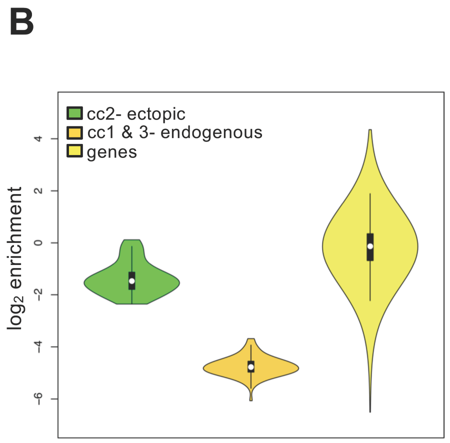

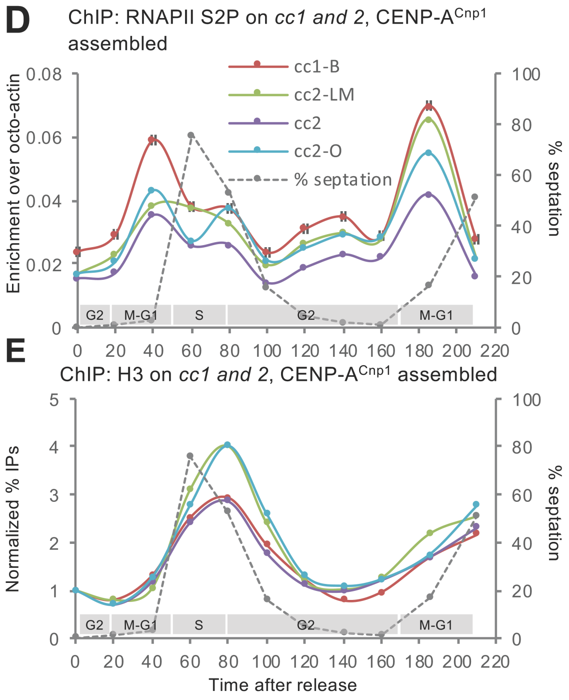

C

RNAPII S2P enrichment	cc1&3	cc2-ectopic	Genes
Maximum	0.07779864	1.08275919	20.3975687
Top 25%	0.04260005	0.45739452	1.27270918
Median	0.03658789	0.36088723	0.91234273
Top 75%	0.03197079	0.28794616	0.62386661
Minimum	0.01491206	0.19569142	0.010989

Figure S5. RNAPII accumulates on centromere DNA in a cell cycle dependent manner. Related to figure 6.

(A) Heat map showing RNAP II S2P enrichment at cell cycle regulated example genes. Enrichments of RNAP II S2P at *ace2*$^{+}$ (M), *adg1*$^{+}$ (G1), *hhf1*$^{+}$ (S), *spd1*$^{+}$ (G2) and *gas2*$^{+}$ (late G2/M) genes are shown corresponding to indicated time points from the ChIP-Seq experiment shown in Figure 6B.

(B) Violin plots of RNAPII-S2P levels over non-centromeric *ura4:cc2* (green) centromeric cc1/cc3 (orange) and genes (yellow) for a representative T0 (G2) sample. Y axis shows log_2 RPKM values for corresponding genomic locations.

(C) Table showing maximum, top 25%, median, top 75% and minimum values for RNAPII-S2P enrichments data shown in (B)

(D) Representative qChIP for RNAPII-S2P levels at endogenous centromeric locations in *cdc25-22* synchronized cell cultures. Y axis: %IP levels were normalized using ChIP levels at *S. octosporus act1*$^+$ from spiked-in chromatin. Septation index and cell cycle phases as indicated.

(E) qChIP for H3 levels from the same cell population as (D). Y axis: %IP levels were normalized first using ChIP levels at *S. octosporus act1*$^+$ from spiked-in chromatin and then to T0 values for each series of samples.

Table S1

List of strains used in the study

Strain no.	Genotype	Reference
FY 1645	*h+ ade6-210 arg3-D4 his3-D1 leu1-32 ura4-D18*	Lab stock
FY 2352	*h+ cdc25-22 ade6-210 leu1-32 ura4-D18*	Lab stock
FY A9169	*h+ ade6-210 cdc25-22 leu1-32 ura4-D18 cnp1:cnp1-loxP-HA-HYG-loxP-T7 ars1:pRAD11-Cre-EBD-leu2+*	This study
FY B2824	*ade6-210 cdc25-22 leu1-32 ura4-D18 cnp1:cnp1-loxP-HA-HYG-loxP-T7 ars1:pRAD13-Cre-EBD-leu2+*	This study
FY B2827	*ade6-210 cdc25-22 leu1-32 ura4-D18 cnp1:cnp1-loxP-T7-HYG-loxP-HA ars1:pRAD13-Cre-EBD-leu2+*	This study
FY B1390	*h+ kanMX6-Pcnp1-mEGFP-cnp1 ade6-M210 leu1-32 ura4-D18 cdc25-22*	This study
FY A9210	*H3.2-lox-T7-HYG-lox-HA ars1:pRAD11-Cre-EBD-leu2+ cdc25-22 ura4D18/DSE leu1-32*	This study
FY B0324	*cc2Δ6Kb:cc1 Ura4-DSE-sup3e-cc2-ura4+ H3.2-lox-HA-HYG-loxT7 ars1:pRAD11-Cre-EBD-leu2+ cdc25-22 ade6-704-NAT ura4D18/DSE leu1-32*	This study
FY 6443	*h+ GFP-hht2 leu1-32 ade6-210*	MB1346. Gift from M. Balasubramanian
FY A8240	*h- kanMX6-Pcnp1-mEGFP-cnp1 ade6-M210 leu1-32 ura4-D18*	JW2595. Gift from Jian-qiu Wu.
FY B3498	*cdc25-22 ade6-210 arg3-D4 leu1-32 ura4-D18 cc1 : arg3*	This study

Table S2

List of primers used in the study

q-cc2-fw	AAACAAACAACGGCACACTG
q-cc2-rev	AAGCCAGCAAATTCCTTGAGT
q-cc2-LM-fw	GCATCTATTGTACTCTCTC
q-cc2-LM-rev	GAAGGATGGATATGCACGT
q-cc2-O-fw	GACTATAACTAGACCACTCAG
q-cc2-O-rev	CTAGATGAATACTCAAGAAAGC
q-cc2-P-fw	CTGCATATTCGACATCTTGAG
q-cc2-P-rev	AGCCTGTCCATCGCAAAAGG
q-cc1-fw	CAGACAATCGTTTCCATTCAG
q-cc1-rev	AGGTGAAGCGTAAGTGAGTG
q-cc1A-fw	GCTAACGAGGCTAACCCACT
q-cc1A-rev	GAGGTTTTTCGTTCTTAGGGCT
q-cc1B-fw	CCGTTGCAACTTACATCAGCA
q-cc1B-rev	CCGGTCGCCAAATAGCAATG
q-act1-fw	GGTTTCGCTGGAGATGATG
q-act1-rev	ATACCACGCTTGCTTTGAG
q-Sp-act1-fw	AGAAGAAATCGCAGCGTTGG
q-Sp-act1-rev	GGTCTACCGACAATCGAGGG
q-So-act1-fw	CAGGTCACCACAGCCTTACA
q-So-act1-rev	CTTGCACATACCAGAGCCGT
q-dg-fw	AATTGTGGTGGTGTGGTAATA
q-dg-rev	GGGTTCATCGTTTCCATTCAG
q-pyk1-5'fw	CTGTCAACCGTCGTACCTCC
q-pyk1-5'rev	TCATACCAGCATCACGGAGC
q-pyk1-GBfw	GTCCGTGTCAACAACAACGG
q-pyk1-GBrev	CTTGACACCGAAGCGGAGAT
q-spd1-5'fw	AGTAGCAAACGCTCCCACAA
q-spd1-5'rev	GGTCATAACTCGCTTGCTGC
q-spd1-GBfw	CTGCCTATAACCCACCGCTT
q-spd1-GBrev	TGCTCGAATGGACGCTTAGT
q-sua1-fw1	TGCTCATTCACCCTGTCGTC
q-sua1-rev1	GGCGGAACCCTTAGGATAGC
q-sua1-fw2	CAGCCTTGAACGAGGATGGT
q-sua1-rev2	AGAAACAACAACGGCAGCAC
q-rpb1-fw	GAAATAGCGGGACAGCCTCA
q-rpb1-rev	TTGCCACGTAATCGACCCTC
q-arg3-P1fw	TGTCGTCTCAGAATGTCGCC
q-arg3-P1rev	GGCAACTGACAGCACTTTCG
q-arg3-P2fw	GCTAAGCCCAAGGACGTCAA
q-arg3-P2rev	TAACAGCAACCTTGGGGTCG
q-arg3-P3fw	TTGTTTACCCCGTCATCCCG
q-arg3-P3rev	TCCATTTGCGGTTCTCTGCT
